# Filamin B Regulates Chondrocyte Proliferation and Differentiation through Cdk1 Signaling

**DOI:** 10.1371/journal.pone.0089352

**Published:** 2014-02-14

**Authors:** Jianjun Hu, Jie Lu, Gewei Lian, Jingping Zhang, Jonathan L. Hecht, Volney L. Sheen

**Affiliations:** 1 Department of Neurology, Beth Israel Deaconess Medical Center and Harvard Medical School, Boston, Massachusetts, United States of America; 2 Department of Pathology, Beth Israel Deaconess Medical Center and Harvard Medical School, Boston, Massachusetts, United States of America; INSERM U1059/LBTO, Université Jean Monnet, France

## Abstract

Humans who harbor loss of function mutations in the actin-associated *filamin B* (*FLNB*) gene develop spondylocarpotarsal syndrome (SCT), a disorder characterized by dwarfism (delayed bone formation) and premature fusion of the vertebral, carpal and tarsal bones (premature differentiation). To better understand the cellular and molecular mechanisms governing these seemingly divergent processes, we generated and characterized *FlnB* knockdown ATDC5 cell lines. We found that *FlnB* knockdown led to reduced proliferation and enhanced differentiation in chondrocytes. Within the shortened growth plate of postnatal *FlnB^−/−^* mice long bone, we observed a similarly progressive decline in the number of rapidly proliferating chondrocytes and premature differentiation characterized by an enlarged prehypertrophic zone, a widened Col2a1^+^/Col10a1^+^ overlapping region, but relatively reduced hypertrophic zone length. The reduced chondrocyte proliferation and premature differentiation were, in part, attributable to enhanced G2/M phase progression, where fewer FlnB deficient ATDC5 chondrocytes resided in the G2/M phase of the cell cycle. FlnB loss reduced Cdk1 phosphorylation (an inhibitor of G2/M phase progression) and Cdk1 inhibition in chondrocytes mimicked the null FlnB, premature differentiation phenotype, through a β1-integrin receptor- Pi3k/Akt (a key regulator of chondrocyte differentiation) mediated pathway. In this context, the early prehypertrophic differentiation provides an explanation for the premature differentiation seen in this disorder, whereas the progressive decline in proliferating chondrocytes would ultimately lead to reduced chondrocyte production and shortened bone length. These findings begin to define a role for filamin proteins in directing both cell proliferation and differentiation through indirect regulation of cell cycle associated proteins.

## Introduction

Mutations in *FLNB* cause two types of skeletal dysplasias on the basis of their clinical presentation and genetic etiology. Null alleles of *FLNB* cause recessive spondylocarpotarsal syndrome (SCT; OMIM 272460), which features dwarfism and fusion of the vertebral, carpal, and tarsal bones. Autosomal dominant mutations of *FLNB* (missense mutations, small in-frame deletions or insertions) cause a group of skeletal dysplasias, including Larsen syndrome (LS; OMIM 150250), atelosteogenesis I and III (AOI and AOIII; OMIM 108720 and 108721), and boomerang dysplasia (BD; OMIM 112310) [1], [2], [3]. LS features joint dislocations, cervical spine malformations, and supernumerary carpal and tarsal ossification centers [1]. AOI, AOIII, and BD exhibit more severe phenotypes including undermodeled bones or ossification initiation failure [2], [3], [4]. A minority of individuals survive in AOIII, while AOI and BD present with fetal/perinatal lethality.

Several studies have described the skeletal phenotypes seen with loss of FlnB mouse models. In general, loss of FlnB function in mice leads to two major skeletal phenotypes, dwarfism, and premature mineralization with bone fusion [5], [6]. We have previously shown a progressive delay and shortening in formation of the long bones, giving rise to the smaller stature seen in the null mice [6]. Other FlnB mutant mice with truncation mutations (at the N-terminal position1624 of the full-length 2602 amino acids) developed early fusion of the spinal vertebrae, due to enhanced chondrocyte hypertrophy and premature differentiation [5], [7], [8]. The mechanisms affecting these two apparently incongruent processes of overall reduction in bone growth and premature differentiation are not clear.

Longitudinal endochondrial bone growth is controlled by the growth plate, which consists of columns of chondrocytes. Chondrocytes progress through division, rotation, pushing forward and differentiation into hypertrophic cells, which are then replaced by osteocytes. In this respect, the rates of cell cycle progression and timing of cell cycle exit will affect long bone growth. Cell cycle progression is mediated by cyclin-dependent kinases (Cdks), their activators, and inhibitors [9], [10]. Multiple cell cycle genes have been implicated in long bone growth, such as Cyclin D1 in affecting G1 phase of cell cycle [11]. Filamins have been implicated in regulation of Cdk1/Cyclin B, which affect the G2/M phase of cell cycle [12]. Moreover, several kinases (Aurora) have been shown to influence cell fate within this same phase of the cell cycle [13], [14]. In this respect, G2 to M phase progression through Cdk1/Cyclin B can potentially influence both cell proliferation and differentiation.

We have previously shown that filamins can regulate cell differentiation and proliferation through their association with cell cycle associated proteins in mouse central nervous system [12]. More specifically, the smaller brain size seen in loss of *Filamin A* (*FlnA*) mice results from a delayed differentiation of neural progenitors and prolongation of the cell cycle in G2/M phase, leading to slower proliferation rates [12]. In this report, we find that loss of FlnB similarly leads to a shortening (albeit of the skeletal long bones), and is also accompanied by a progressive decline in the number of proliferating chondrocytes over time. However, instead of delayed differentiation and a prolongation of the cell cycle in G2/M phase giving rise to this shortening, as seen with FlnA inhibition, *FlnB* knockout (*FlnB* ^−/−^) mice show an increase in early onset differentiation within the lower proliferative and prehypertrophic zones, and this maturation is associated with fewer rapidly proliferating chondrocytes. Fewer proliferating null FlnB chondrocytes remain in the G2/M phase, suggestive of either fewer cells entering this phase or more rapid progression through G2/M. Similarly, enhanced chondrocyte maturation and a reduction in proliferative rates are seen in stably transfected ATDC5 chondrocytes lacking in FlnB. Loss of FlnB in the ATDC5 cells further causes a downregulation of the G2/M phase inhibitor phospho-Cdk1, and inhibition of phopho-Cdk1 promotes G2/M phase progression and chondrocyte differentiation. Finally, we show that FlnB can modulate Cdk1 phosphorylation through a β1 integrin-Pi3k/Akt dependent pathway. These findings suggest that loss of FlnB leads to premature chondrocyte differentiation into the prehypertrophic state. Although prehypertrophic chondrocytes continue to proliferate, the increased maturity likely slows chondrocyte proliferative rates, and produces fewer chondrocytes over time, thereby producing the observed phenotypes of both dwarfism and early differentiation.

## Results

### Decline in Chondrocyte Proliferation and Increased Differentiation with FlnB Knockdown in vitro

Given the difficulty in studying primary chondrocyte cultures, we generated several stable ATDC5 chondrocyte cell lines with reduced *FlnB* expression through shRNA knockdown. Two target sequences were designed (FlnBsh1 and FlnBsh2) and their expression levels were gauged by EGFP tagged lentiviral production and infection into ATDC5 cells (Supplementary Material, Fig. S1A). Lines generated from several FlnA targeting sequences and non-targeting sequences served as controls. Infection with lentivirus carrying either shRNA sequence led to downregulation of FlnB expression at both the mRNA and protein levels (Supplementary Material, Fig. S1B). Both FlnBsh1 and FlnBsh2 caused down-regulation of the premature chondrocyte marker, Sox9, levels and up-regulation of the chondrocyte differentiation marker, Runx2, in ATDC5 cells (Supplementary Material, Fig. S1B). The FlnBsh2 sequence showed a higher knockdown efficiency than FlnBsh1, and was therefore used primarily to display the results from subsequent experiments, although similar results were obtained from both lines. No changes in cell death were seen in established cell lines.

**Figure 1:**
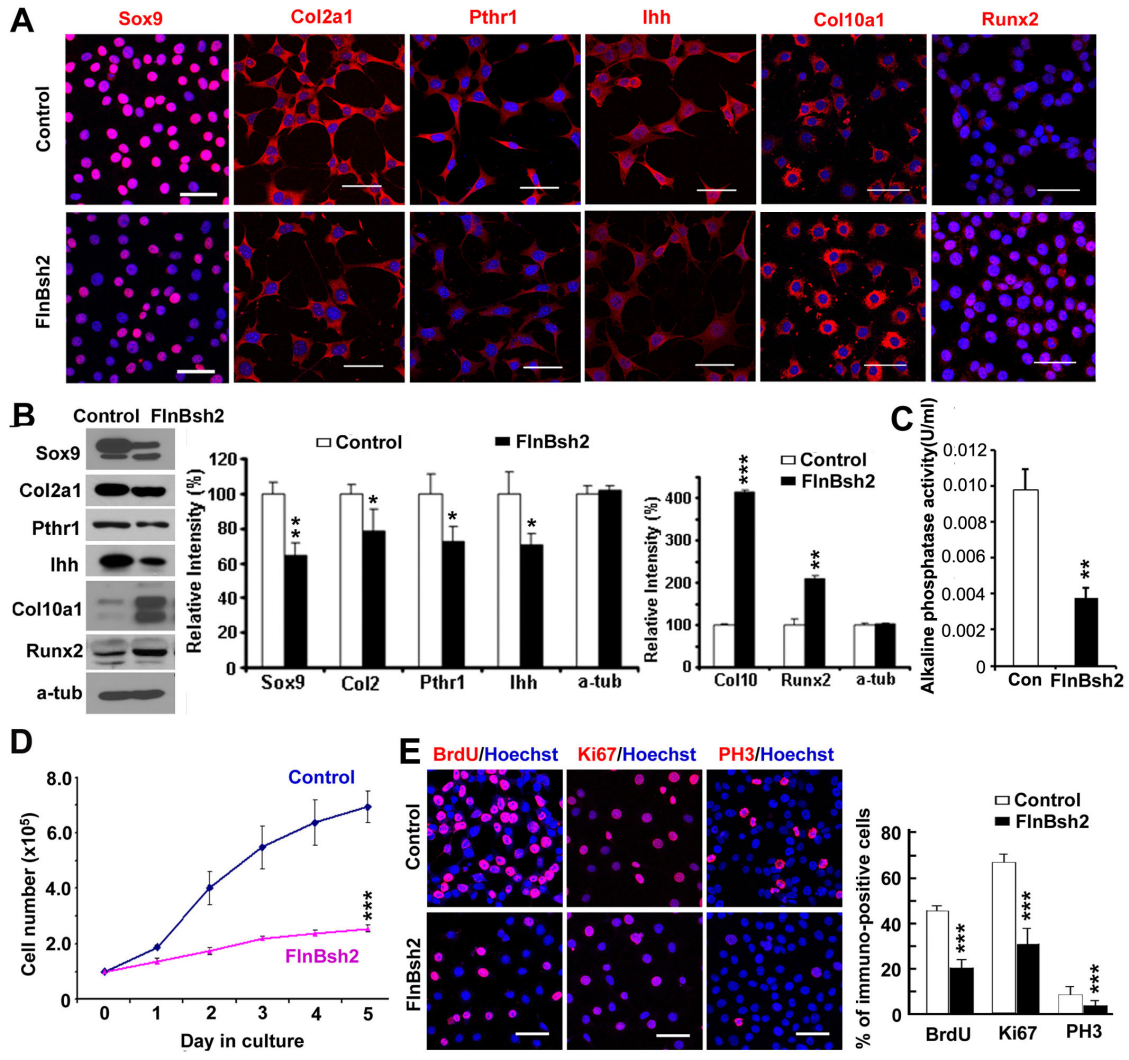
Reduced proliferative capacity and enhanced differentiation in FlnB knockdown proliferating chondrocytes. (A) Fluorescent immunocytochemistry performed on FlnB knockdown ATDC5 (FlnBsh2) chondrocytes shows downregulation of both proliferating chondrocyte markers, Sox9 and Col2a1 in the FlnB knockdown ATDC5 cells. The immunostaining also shows down-regulation of the prehypertrophic markers, Pthr1 and Ihh in the FlnB knocked down cells and increased expression for the hypertrophic marker Col10a1 and chondrocyte differentiation marker Runx2 in FlnBsh2 proliferating chondrocytes. (B) Western blot analyses of FlnBsh2 chondrocytes show a similar increased differentiation of the ATDC5 chondrocytes following FlnB knockdown compared to control. Results are graphically summarized to the right. (C) Alkaline phosphatase assay. Alkaline phosphatase activity was decreased in FlnB knockdown ATDC5 cells. (D) FlnBsh2 chondrocytes undergo a slower growth rate compared to normal ATDC5 cells. Quantification reveals an approximate 64% reduction in the proliferation rate by 5 days in culture. Cells are plated at a density of 1×10^5^ and quantified at daily intervals. (E) Statistical analyses (n≥3 independent samples per experiment) show that the numbers of cells per unit area positively labeled for BrdU, Ki67, and PH3 (red) FlnBsh2 proliferating chondrocytes are decreased by 57%, 46%, and 45%, respectively when compared to control ATDC5 cells. * = p<0.05, ** = p<0.01, *** = p<0.001. Scale bars = 50 µm.

To better understand the role of FlnB knockdown in effecting chondrocyte differentiation, we examined the expression levels of various chondrocyte markers for the proliferative (Collagen2 (Col2a1) and Sox9 [15], [16]), prehypertrophic (parathyroid hormone receptor 1 (Pthr1) and Indian hedgehog (Ihh) [17], [18], [19]), and differentiation/hypertrophic zones(Collagen 10 (Col10a1) [20], [21] and Runx2 [22], [23]). Sox9 and Col2a1 levels were decreased (35.8% and 21.8%, respectively, versus control), while the hypertrophic differentiation marker Col10a1 and the hypertrophic-required transcription factor Runx2, were increased (314.8% and 110.4%, respectively, versus control) in FlnBsh2 cells as assessed by immunocytochemistry and western blot analyses (Fig. 1A, B). The intermediate prehypertrophic markers, Pthr1 and Ihh, were also decreased in the loss of FlnB cells (27.9% and 29.3%, respectively, versus control), suggesting enhanced differentiation with prolonged culturing *in vitro*. To further characterize the differentiation, we performed alkaline phosphatase assay and found that the alkaline phosphatase activity was decreased but not increased, which suggested that FlnB loss promoted some but not all features of hypertrophic chondrocyte differentiation (Fig. 1C).

To address the effect of FlnB loss on chondrocyte proliferative rates, we plotted the growth curves for the respective cell lines and used several proliferation markers (immunostaining of BrdU, Ki67 and phosphohistone H3 (PH3)). We observed a significant reduction in the proliferative capacity (as gauged by cell number) of the FlnBsh2 cultures over time (Fig. 1D). Approximately 20% of FlnB knockdown cells incorporated BrdU as compared to 45% in control ATDC5 cells after 1-hour incubation. Ki67 staining and quantitative analyses showed that only 31% of FlnB knockdown ATDC5 cells were Ki67^hi^ compared with 67% in the control group. Lastly, PH3 staining and analyses showed a similar trend with 3.9% of the FlnBsh2 cells staining positive for the M phase marker, compared with 8.7% in control (Fig. 1E). Collectively, these results suggest that FlnB loss impairs chondrocyte proliferation while simultaneously promoting differentiation.

### Loss of FlnB Leads to Postnatal Long Bone Shortening Accompanied with Prehypertrophic Zone Widening

Previously we had focused on defects in chondrocyte migration giving rise to a delay in bone growth, leading to shortened long bones in embryonic *FlnB^−/−^* mice [6]. However, these observations of delayed skeletogenesis would seemingly be inconsistent with the premature chondrocyte differentiation seen in FlnB knockdown ATDC5 cells. We therefore examined the long bone development in early postnatal *FlnB^−/−^* mice to ascertain whether changes in chondrocyte development over time could reconcile these differences. Consistent with our prior characterization of embryonic *FlnB^−/−^* mice, we observed a similar shortening of the appendicular long bones in postnatal *FlnB^−/−^* mice (P1 to 8 weeks; quantification analysis of 4 weeks and 8 weeks are shown in Supplementary Material, Fig. S2A). The bone length was reduced by approximately 1.4 mm at P1 and up to 4 mm by 8 weeks. The length of the growth plate was significantly shortened in *FlnB^−/−^* mice compared to age-matched controls (Supplementary Material, Fig. S2B). Lastly, the ratio of Col10a1 to Col2a1 was also decreased in the loss of FlnB postnatal mice, suggestive of an overall delay (Supplementary Material, Fig. S3A, B). Overall, these findings were consistent with a delay in skeletogenesis (previously observed in the embryonic *FlnB^−/−^* mice) and in contrast with the early differentiation seen with FlnB knockdown ATDC5 chondrocytes.

**Figure 2:**
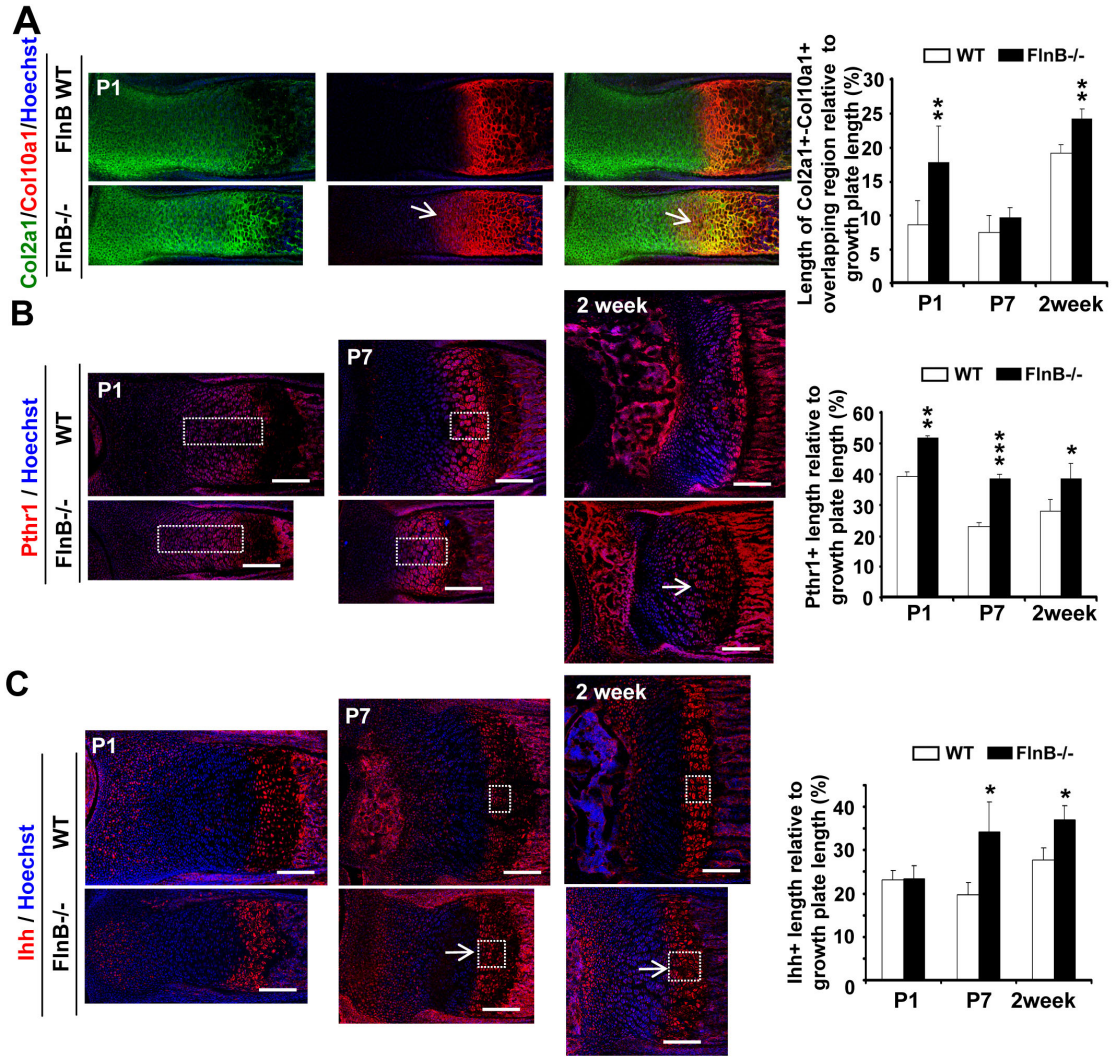
Premature differentiation within the prehypertrophic zone in the long bone growth plates with loss of FlnB function. (A) Col2a1 and Col10a1 double immunostaining on P1 null FlnB radius shows an abnormally increased region of overlapping expression for the proliferation and hypertrophic markers (open arrow). Quantitative analysis of the length of Col2a1^+^-Col10a1^+^ overlapping expression relative to the growth plate length. The Col2a1^+^-Col10a1^+^ overlapping region is increased in FlnB^−/−^ mice at P1 and 2 week old age (17.7% vs 8.6%, 24.2% vs 19.1% in FlnB^−/−^ and wild type, respectively). The ratio at P7 shows the similar, albeit not significant, trend of change. At P1, n samples = 6, for P7 and 2 week age, n samples = 3. (B) Pthr1 antibody immunostaining. Pthr1^+^ zones are thickened in the FlnB^−/−^ radius at P1, P7 and 2 weeks. At 2 weeks, most of the chondrocytes become Pthr1^+^ in some FlnB^−/−^ mice (open arrow). (C) Ihh antibody immunostaining. Ihh^+^ zones are thickened in the FlnB^−/−^ radius at P1, P7 and 2 weeks. At 2 weeks, the Ihh^+^ zone covers both the prehypertrophic and hypertrophic zones in most of the FlnB^−/−^ mice (open arrow). Scale bars = 200 µm.

Changes in the rate of differentiation as chondrocytes progress through the proliferative, prehypertrophic and hypertrophic zones could potentially reconcile the precocious maturation but delay in skeletal development, seen in chondrocytes with loss of FlnB function. We therefore examined the expression of several chondrocyte markers [24], including Sox9 and Col2a1 (less differentiated/proliferative zone), Pthr1 and Ihh (intermediate differentiation/prehypertrophic zone), Runx2 and Col10a1 (more differentiated/hypertrophic zone) across different zones in the growth plate of the long bones in postnatal mice. In the postnatal null FlnB mice, we detected abnormally increased expression of the hypertrophic markers Col10a1 (Supplementary Material, Fig. S3A, C). Quantitative analysis of the Col10a1^+^ length/growth plate length ratio at P1 age showed an 10% increase of the relative width of the hypertrophic zone (38.7% in Flnb^−/−^ vs 28.5% in wild type), which suggested that chondrocytes were undergoing premature hypertrophic differentiation in FlnB^−/−^ long bone (Supplementary Material, Fig. S3C). This trend toward early maturation was seen at P7 and 2 weeks but was not statistically significant suggesting a slowing of this process over time. Additionally, fewer Sox9^+^ labeled chondrocytes were appreciated with loss of FlnB (47.8% in FlnB^−/−^ vs 81.4% in wild type; Supplementary Material, Fig. S4A) and increased signal intensity for Runx2 was observed in the FlnB^−/−^ mice at P7 age (32.6 in FlnB^−/−^ vs 28.9 luminosity in wild type; Supplementary Material, Fig. S4B). These analyses were in contrast with observations of the decreased Col10a1^+^ length relative to Col2a1^+^ length (suggestive of delayed differentiation). This difference could potentially be accounted for by the increase in overlap between Col10a1 and Col2a1 in the loss of FlnB mice (Fig. 2A, and Supplementary Material, Fig. S3A), indicating that more proliferative zone chondrocytes (Col2a1^+^) were undergoing proliferative to prehypertrophic transition. Immunostaining of the growth plates with Pthr1 and Ihh demonstrated an increase in the prehypertrophic zone in FlnB^−/−^ mice (Fig. 2B and C). The ratio of Pthr1^+^ length to growth plate length, was increased (51.7% vs 39.2%, 38.7% vs 23.0% and 38.6% vs 28.1%, in *FlnB^−/−^* vs wild type at P1, P7 and 2 weeks, respectively, Fig. 2B). Quantitative analysis showed that the ratio of Ihh^+^ length/growth plate length, was increased (34.2% vs 19.6% and 37.0% vs 27.7%, in *FlnB^−/−^* and wild type, at P7 and 2 week old age, respectively, Fig. 2C). Both the increase in Col2a1^+^-Col10a1^+^ overlapping expression and the increase in Pthr1^+^ and Ihh^+^ zone would suggest that loss of FlnB promotes premature prehypertrophic chondrocyte differentiation and/or impairs transition from the prehypertrophic to hypertrophic zone.

### Decline in Chondrocyte Proliferation and Increased Entry of Chondrocytes into the Resting State with Loss of FlnB Function in vivo

**Figure 3:**
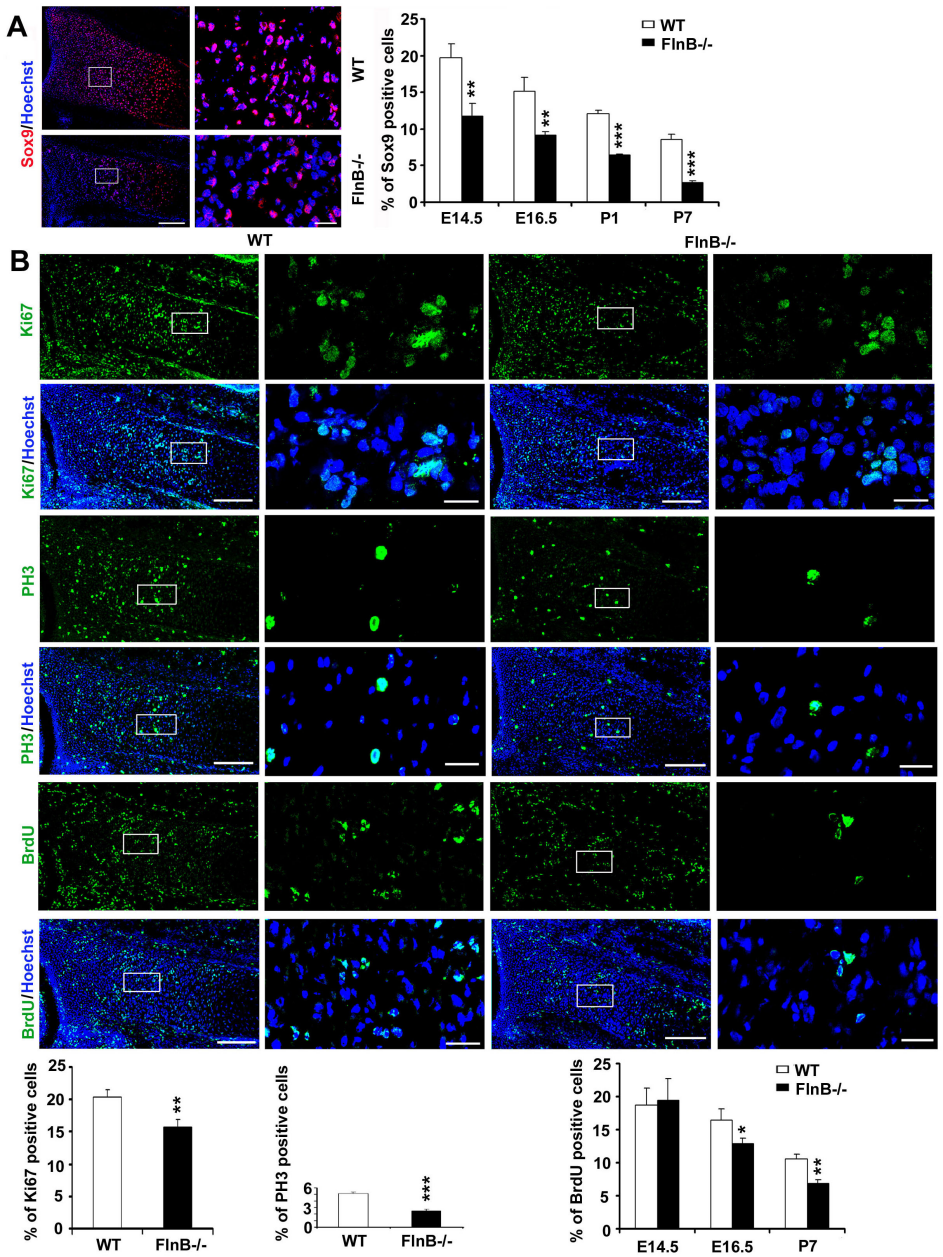
FlnB^−/−^ mouse chondrocytes display decreased proliferation at postnatal ages. (A) Immunostaining for Sox9 shows a decrease in the proportion of Sox9+ cells in growth plates of FlnB^−/−^ mice at all detected ages. The data are quantified and graphically summarized to the right. (B) Immunofluorescent photomicrographs of E16.5 FlnB^−/−^ and control radial bone growth plate after immunostaining for proliferation markers: BrdU (fluoroscein), a marker labeling cells entering into S-phase; Ki67 (fluoroscein), a marker for cells in the cell cycle; and phospho-histone H3 (PH3) (fluoroscein), an M-phase marker. Higher magnification images of the outlined boxes are to the right. There is an overall reduction in each of these markers within the proliferative zone after FlnB knockout at various ages. Statistical analyses (n≥3 independent samples per experiment) shows that the percentage of cells positively labeled for Sox9, BrdU, Ki67, and PH3 in *FlnB^−/−^* cortex is decreased, respectively when compared to littermate controls. * = p<0.05, ** = p<0.01, *** = p<0.001 by t-test. Scale bar = 200 µm for low magnification; 50 µm for high magnification.

In general, a decrease in proliferation or increase in cell death could explain bone shortening due to a fewer number of chondrocytes. To address this possibility, we used markers for cell proliferation (Sox9 and Ki67 for proliferating cells, BrdU for cells in S-phase, and phospho-histone H3 (PH3) for cells in M-phase). Immunostaining was performed on the radial bones of *FlnB^−/−^* mice at various ages, ranging from mid-embryonic (E14.5) to 7 days postnatal (P7). There was a progressive reduction in Sox9, BrdU, Ki67, and PH3 staining over time in *FlnB^−/−^* mice (Fig. 3). Although we did not see a significant difference between WT and FlnB^−/−^ mice in our prior work [6] using BrdU labeling at the E16.5 time point, we had observed a trend in a decrease in the number of proliferating chondrocytes. This trend became significant with increased sample numbers at this time point and even more apparent at older postnatal ages. Although we observed increased apoptosis along the periphery of the hypertrophic zone along the perichondrium [6], no increase in cell death was observed by TUNEL staining in the growth plates in proliferation zone during these ages (data not shown). Overall, these findings began to suggest that a reduction in proliferating chondrocytes could be responsible for a reduction in long bone length and growth.

**Figure 4:**
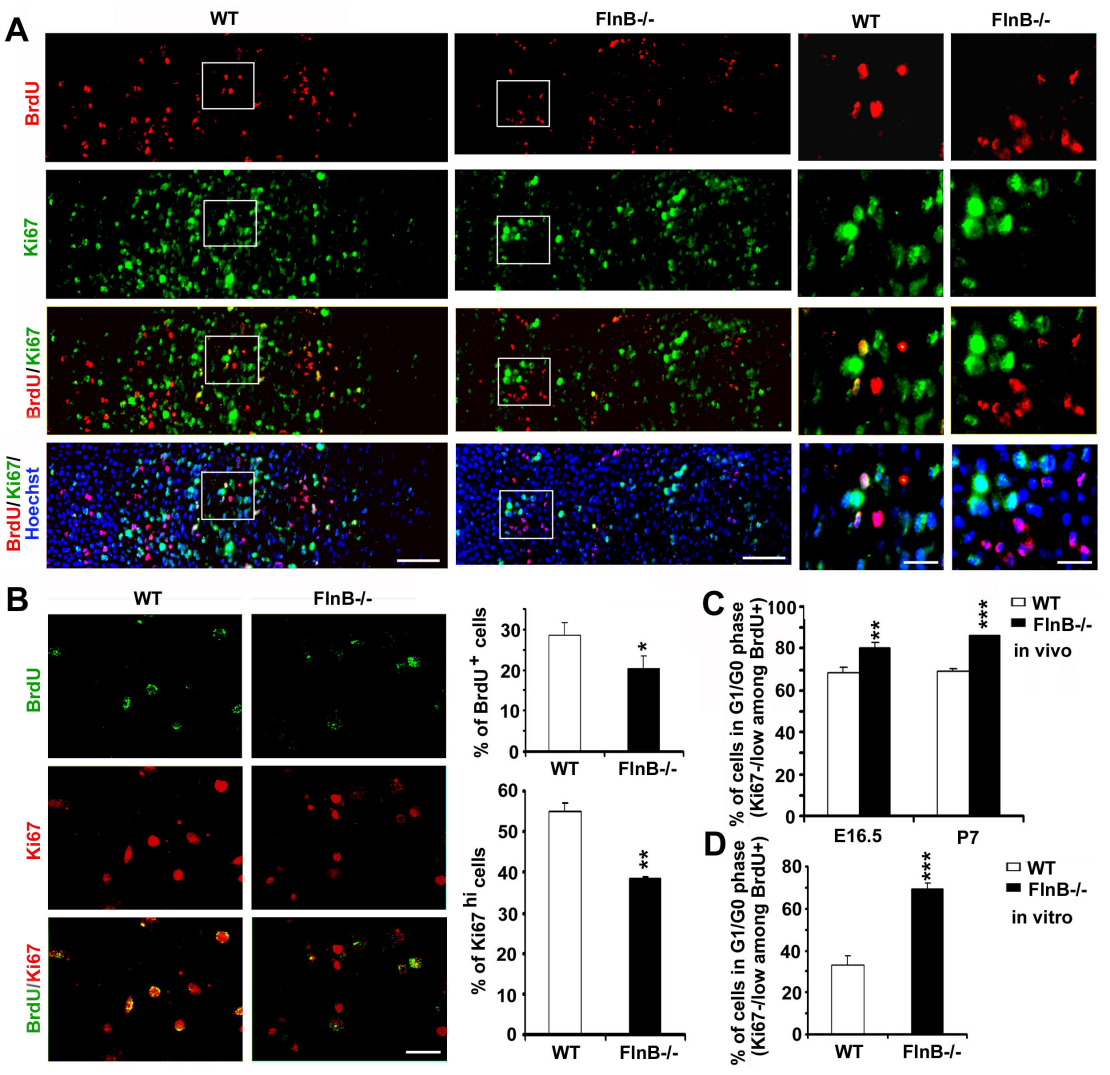
Increased proliferating chondrocytes remaining in G1/G0 phase in null FlnB. (A) Co-staining for Ki67 (fluoroscein) and BrdU (rhodamine) *in vivo* demonstrates an increase in the number of proliferating chondrocytes in the *FlnB^−/−^* radius that remain in G1/G0 phase, consistent with an increased rate of differentiation. BrdU is given as a single pulse to capture the proliferating progenitors. After 48 hours, the animal is sacrificed and co-staining is performed with Ki67 (a marker of all proliferating cells). BrdU positive progenitors, which remain as progenitors will be Ki67 positive (Ki67^hi^), whereas those are undergoing differentiation (remain in G1 phase) or having been differentiated (remain in G0 phase) will be Ki67 weak/negative (Ki67^−/low^). Higher magnification photomicrographs of the boxed images are shown to the right and demonstrate the reduction in BrdU^+^ proliferating chondrocytes, which remain Ki67^hi^ after 48 hours with loss of FlnB. (B) Cultured primary null FlnB proliferating chondrocytes show a similar reduction in levels of BrdU (fluoroscein) and Ki67 (rhodamine) immunolabeling. The FlnB null proliferating chondrocytes from the culture studies also show an increase in the number of cells that remaining in G1/G0 phase (BrdU^+^, Ki67^−/low^), similar to that seen *in vivo*. (C) and (D) Quantification of the chondrocytes remaining in G1/G0 phase *in vivo* and *in vitro*. The analyses show an increase of approximately 10% and 13% of BrdU^+^ FlnB null chondrocytes remaining in G1/G0 phase in the E16.5 and P7 radial bone, respectively (C) and an increase of approximately 36% of BrdU^+^ FlnB null chondrocytes remaining in G1/G0 phase *in vitro* (D). * = p<0.05, ** = p<0.01, *** = p<0.001. Scale bar = 200 µm for low magnification and 25 µm for high magnification in A; 50 µm in B.

Premature differentiation within the prehypertrophic zone should promote bone formation and would therefore not explain the reduction in bone size. However, increased differentiation might lead to slower chondrocyte proliferation rates and an overall progressive delay in ossification (should it affect the number of proliferating chondrocytes generated over time). To address this possibility, we asked whether a greater proportion of proliferating chondrocyte remained in G1 state, suggestive of chondrocytes adopting a more differentiated state *in vivo*. Lengthened G1 phase has been shown to indicate differentiation status in proliferating cells [25]. One measure of cells in proliferation versus differentiation can be made through dual labeling studies using Ki67 and BrdU. Pulsed injection of BrdU labels proliferating chondrocytes undergoing cell division at a particular instance in time. Secondary labeling with Ki67 at 48 hours post BrdU injection in chondrocyte development captures a subset of BrdU^+^ cells that remain actively proliferating chondrocytes, as opposed to the Ki67^−/low^ (weakly stained) and BrdU^+^ chondrocytes that have adopted more differentiated states. In theory, all the chondrocytes prior to the hypertrophic zone continue to proliferate. However, intense nuclear Ki67 labeling has been shown to correlate with cells in S to M phase, and weak/negative Ki67 expression (Ki67^−/low^) corresponds to cells in G1/G0 phase. The greater numbers of cells that have progressed through S/G2/M phase (BrdU^+^) but remain in G1 phase (weak Ki67 labeling) corresponds to enhanced differentiation. In this context, we observed a greater proportion of BrdU^+^ and Ki67^−/low^ labeled cells in the radial bones of *FlnB^−/−^* mice (89% in *FlnB^−/−^* vs 69% in wild type at E16.5, and 86% in *FlnB^−/−^* vs 69% at P7), indicating again an increase in the number of proliferating chondrocytes which had remained in G1 phase and presumably adopted a more differentiated state (Fig. 4A, C). A similar decline in the number of proliferating chondrocytes in S/G2/M phase was seen in culture, when assessed by BrdU and Ki67. Additionally, a similar trend toward an increased fraction of proliferating chondrocyte cells remaining in G1 phase (BrdU^+^ and Ki67^−/low^) was observed with cultured FlnB knockout chondrocytes (Fig. 4B, D). Taken in the context of increased immunostaining for prehypertrophic markers, these studies suggest that loss of FlnB led to an increased number of actively proliferating chondrocytes residing in the G1 state and therefore adopting more differentiated states.

### Loss of FlnB Promotes G2/M Phase Progression and Down-regulation of Cyclin B Associated Proteins

**Figure 5:**
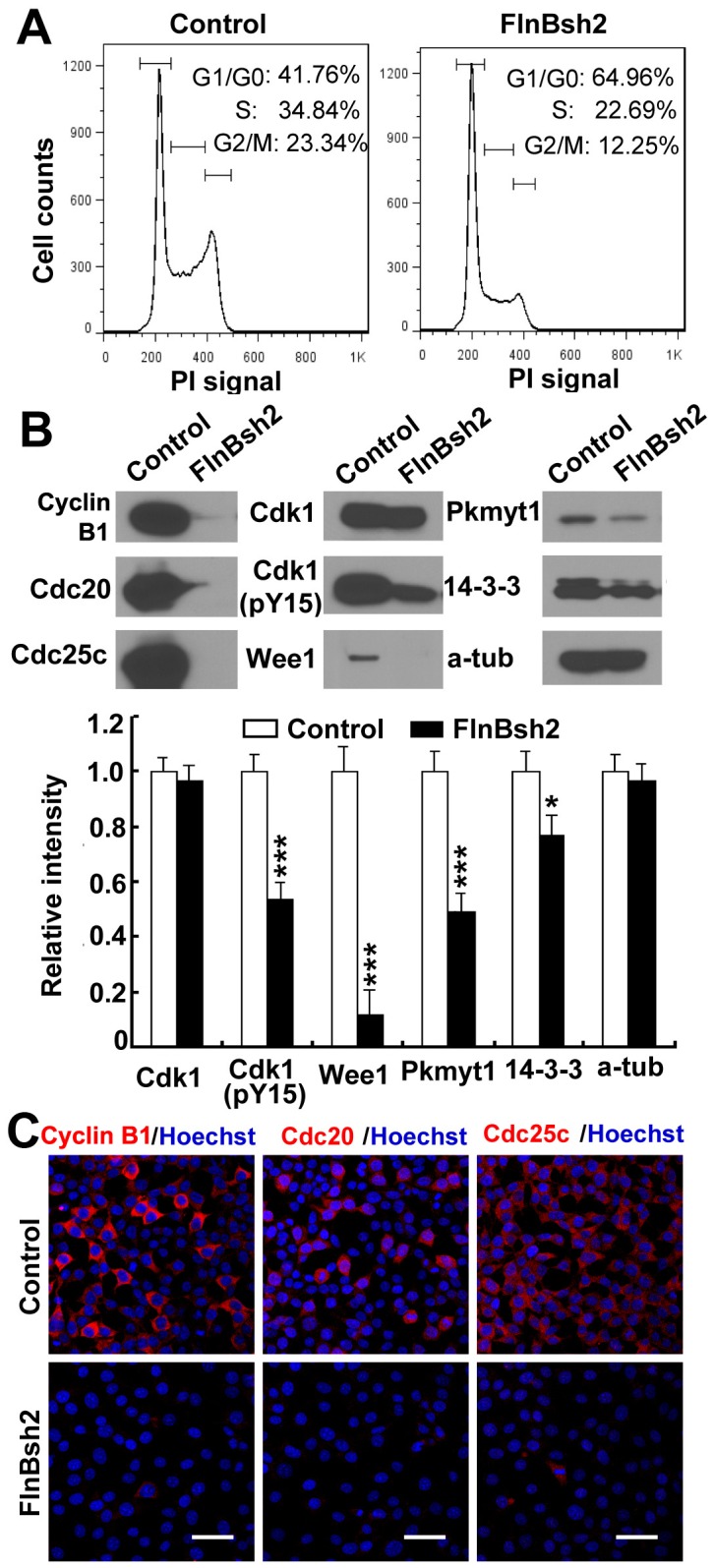
Dysregulation of Cyclin B-associated proteins with loss of FlnB function. (A) Flow cytometry following propridium iodide (PI) labeling demonstrates a decrease in the number of FlnBsh2 chondrocytes that reside in G2/M phase and an increase in the number of cells that reside in G1/G0 phase, compared to ATDC5 control cells. (B) Western blot analyses similarly shows a reduction in these Cyclin B1-associated markers, including Cyclin B1, Cdc20, Cdc25c, Pkmyt1, 14-3-3, and Wee1, within the FlnB knockdown ATDC5 cells. Cdk1 levels are largely unchanged but Cdk1 phosphorylation (pY15) is diminished. G2/M phase progression is mediated by Cdk1 activation (phosphorylation) through the Cyclin B1-associated proteins. Reduced Cdk1 phosphorylation promotes progression through G2/M phase. Changes in Western blot intensity for various proteins expression are quantified below. (C) A corresponding decrease in both the rhodamine fluorescence intensity and number of labeled FlnBsh2 proliferating chondrocytes is seen using various G2/M phase markers, including Cyclin B1, Cdc20, and Cdc25c (rhodamine). Additional markers are shown in supplementary figure S5. * = p<0.05, *** = p<0.001. Scale bar = 50 µm in C.

One possible mechanism that could account for the reduction in proliferating chondrocytes and early differentiation would be a role for FlnB in regulating the cell cycle, similar to that for FlnA [12]. Loss of FlnA led to an increase in neural progenitors in G2/M (and to a lesser degree in G1/S) phase, due to prolonged cell cycle (through Cdk1). Cell cycle prolongation would result in reduced proliferation and a decrease in the number of progenitors/proliferating cells generated over time. In this setting, however, increased cell cycle length would also lead to a delay in differentiation. Flow cytometric analysis of the cell cycle by using propidium iodide (PI) staining showed that, with *FlnB* knockdown, both the S phase and the G2/M phase subpopulations decreased by approximately 12% and 11% (less proliferating chondrocytes), respectively, while the G1/G0 phase subpopulations increased by approximately 23% (more differentiating/differentiated cells) (Fig. 5A). Given the greater change seen in G2/M phase, we then focused on Cyclin B-associated regulators of cell cycle. Both western blotting and immunostaining results showed a decrease in Cyclin B1 expression, which would promote G2/M phase progression (Fig. 5B, C). To transit through the various phases of mitosis, proliferating chondrocytes entering metaphase must first activate Cdk1 (through dephosphorylation of Cdk1) to initiate degradation of Cyclin B1 through the Anaphase promoting complex (APC) system. Progression from metaphase to anaphase and anaphase to G1 is regulated by activators of the APC, such as Cdc20. However, expression of the APC regulator Cdc20 was decreased in the FlnB knockdown cells, and would therefore inhibit Cyclin B1 degradation (and therefore hinder G2/M phase progression). Rather, phospho-Cdk1(pY15) levels were also decreased in the FlnBsh2 cell line, whereas total Cdk1 levels were unchanged, suggesting a role for FlnB in Cdk1 regulation. To clarify the molecular mechanism by which FlnB regulated Cdk1(Y15) phosphorylation, we next addressed whether loss of FlnB altered the expression levels of several inhibitors and activators of Cdk1 activity. In direct contrast to FlnA loss of function in neural progenitors, inhibitors of Cdk1 activation (enhanced phosphorylation) Wee1, Pkmyt1, and 14-3-3 were all significantly downregulated as assessed by immunocytochemistry and western blot analyses of the FlnBsh2 cells (Fig. 5B, C and Supplementary Material, Fig. S5). Cdc25c levels, however, were also down-regulated which should inhibit Cdk1 dephosphorylation (leading to activation), suggesting that FlnB may not play a direct role in modifying the activation/inactivation status of these proteins. Overall, these observations implied that FlnB loss promoted Cyclin B1 degradation by inhibiting Cdk1(Y15) phosphorylation in proliferating chondrocytes, presumably through some upstream pathway.

### Cyclin B Associated Cdk1 Inhibition Reproduces the Loss of FlnB Phenotypes in vitro

**Figure 6:**
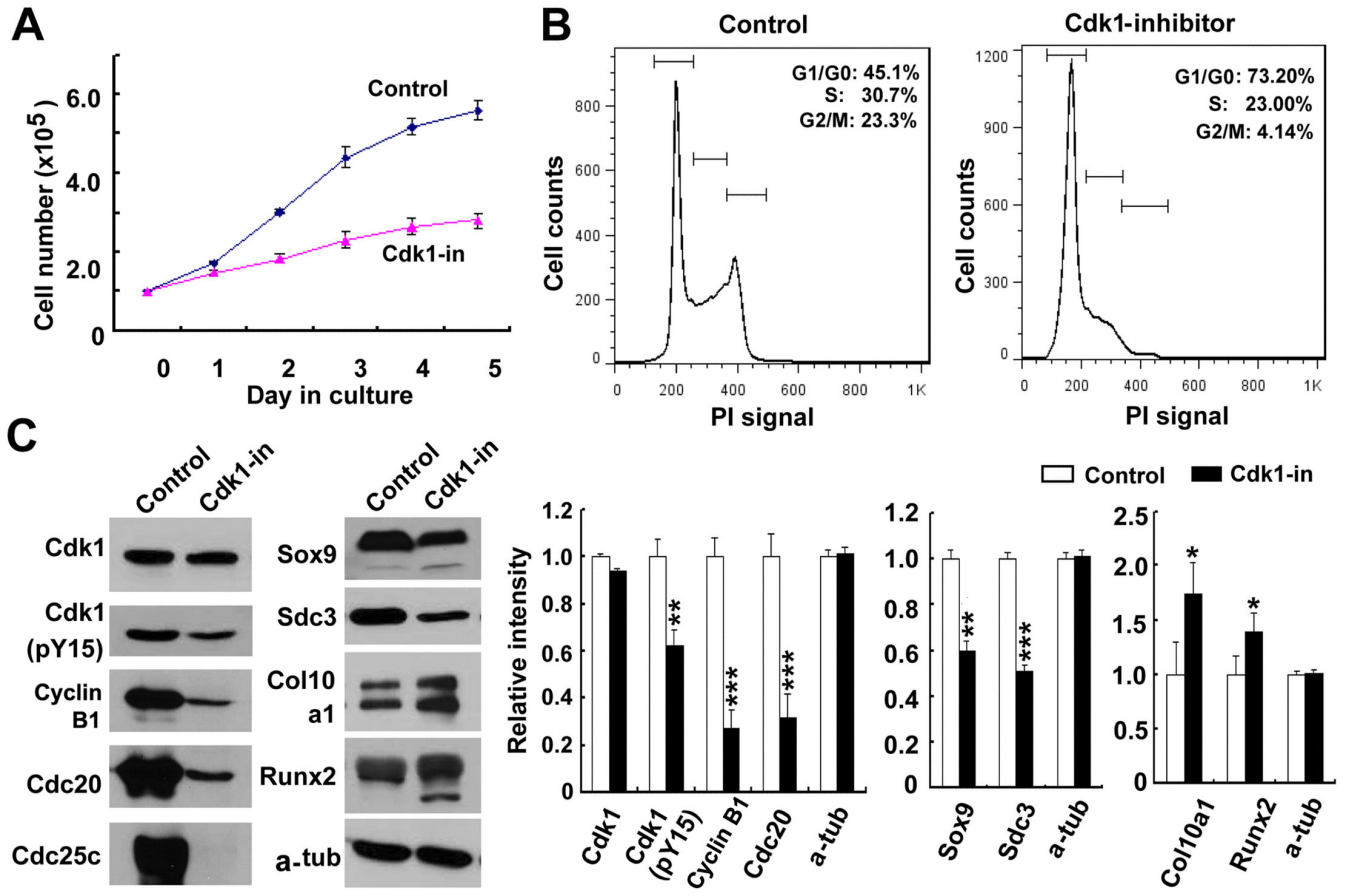
Cdk1 inhibition reproduces the loss of FlnB phenotypes. (A) Following inhibition of Cdk1 activity, ATDC5 progenitors undergo a slower growth rate compared to untreated ATDC5 controls. Proliferation capacity is dramatically decreased at low concentrations of Cdk1 inhibitor (1 µM) and in the absence of any observed increase in cell death. (B) Flow cytometry following propridium iodide (PI) labeling shows a decrease in the number of ATDC5 cells in G2/M phase and an increase in the number of ATDC5 cells in G1/G0 phase in the Cdk1 inhibitor (1 µM) treated cells compared to control. (C) All the cell cycle markers, Cdk1(pY15), Cyclin B1, Cdc20 and Cdc25c, are downregulated following Cdk1 inhibitor treatment, with the exception of total Cdk1 protein levels. Quantification graph is shown to the right. As seen with loss of FlnB function, inhibition of Cdk1 activity (phosphorylation) leads to a decline in early progenitor markers Sox9 and Col2a1, and upregulation of hypertrophic markers Col10a1 and Runx2. Quantitative changes are shown graphically to the right. * = p<0.05, ** = p<0.01, *** = p<0.001.

In order to confirm the role of Cdk1 in loss of FlnB phenotypes, we examined ATDC5 chondrocyte proliferation and differentiation following Cdk1(Y15) inhibition. Exposure of ATDC5 cultures to as low as 1.0 µM phospho-Cdk1 (pCdk1) inhibitor (3-(2-Chloro-3-indolylmethylene)-1,3-dihydroindol-2-one) [26] led to a significant decrease in proliferation rate over 5 days (Fig. 6A). No increase in cell death was observed at this low dosage of Cdk1 inhibitor (data not shown). Next, cell cycle analyses using flow cytometry showed that both active S phase and G2/M phase subpopulations decreased by approximately 8% and 19% (less proliferating chondrocytes), respectively, following treatment with the Cdk1 inhibitor, while the G1/G0 phase subpopulations increased by approximately 28% (more differentiating/differentiated cells)- much in the same manner and distribution as seen with knockdown of FlnB (Fig. 6B). Further analysis of the cell cycle markers also showed that all the cell cycle markers, Cdk1(pY15), Cyclin B1, Cdc20 and Cdc25c, were downregulated, except that total Cdk1 protein levels were comparable (Fig. 6C). Cdk1(pY15), Cyclin B1, and Cdc20 were decreased by about 40%, 75% and 70%, respectively. Cdc25c levels were not quantified because of its very low expression in FlnB knockdown groups. Cdk1 phosphorylation inhibition led to a downregulation of proliferating chondrocyte markers Col2a1 and Sox9 with ATDC5 chondrocytes adopting expression of markers associated with a more differentiated chondrocyte state (Col10a1 and Runx2) (Fig. 6C). Col2a1 and Sox9 were decreased by about 30% and 35%, while Col10a1 and Runx2 were increased by about 75% and 40%, respectively.

### Pi3k/Akt Pathway Contributes to the Cdk1 Activity Changes in vitro through β1 Integrin

**Figure 7:**
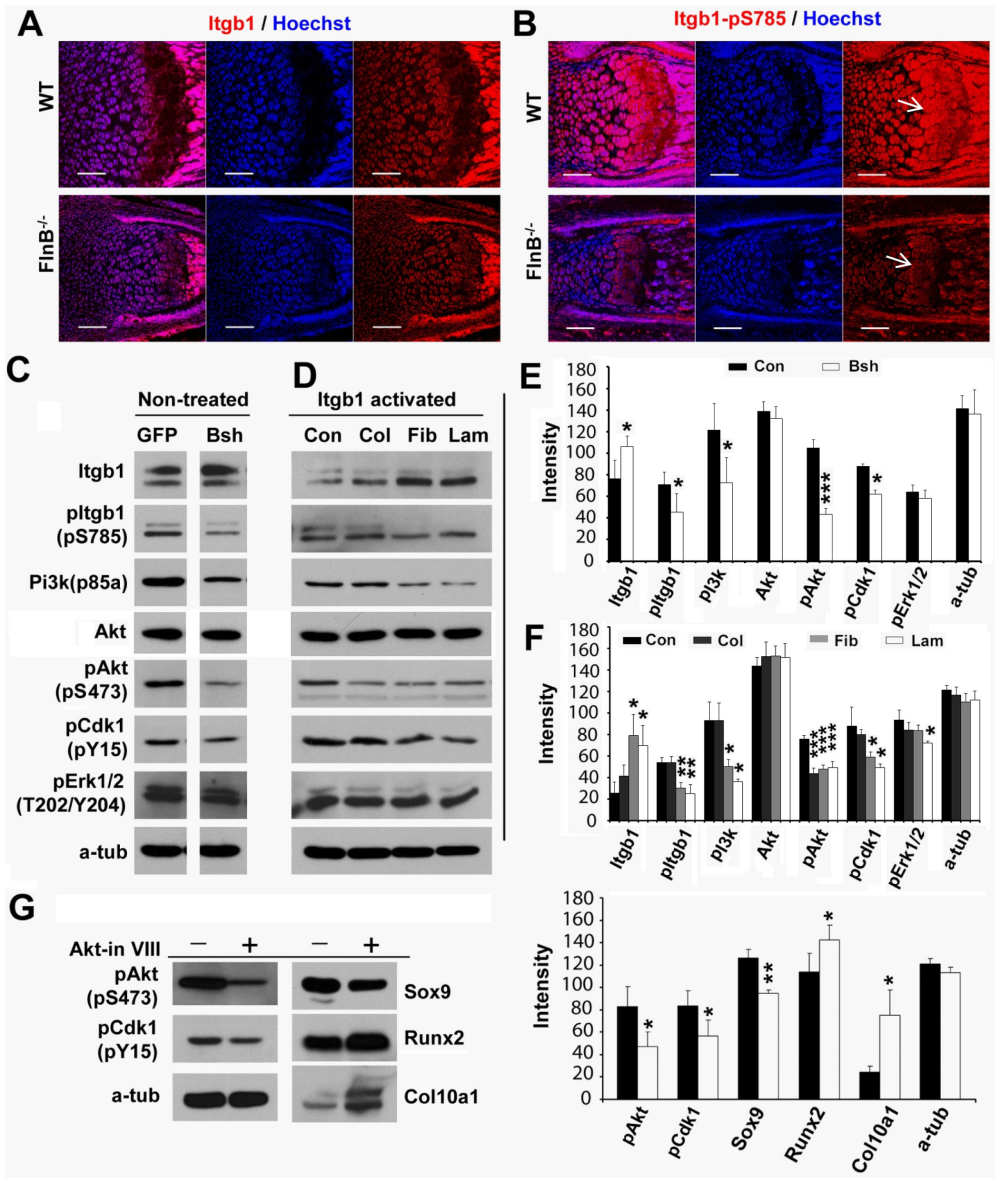
Loss of FlnB induces Cdk1 activity changes through β1 integrin-Pi3k/Akt pathway. (A, B) Immunostaining of total and phospho-β1 integrin (pS785)(postnatal day 1 radius). Phospho-β1 integrin (pS785) levels are down-regulated in FlnB knockout chondrocytes (arrows) (B). (C, E) Western blotting results show that Pi3k(p85 subunit) and phospho-Akt(pS473), as well as phospho-Cdk1(pY15) are down-regulated in FlnB knockdown (Bsh) ATDC5 cells. Total Akt levels are not changed. Total β1 integrin levels are up-regulated but phospho-β1 integrsin (pS785) are down-regulated. Results are quantified in (E). (D, F) β1 integrin activation (Itgb1) in ATDC5 cells regulates Pi3k/Akt and Cdk1 activation. Pretreatment of ATCD5 cells with fibronectin and laminin I but not collagen (col) induces up-regulation of total β1 integrin levels but down-regulation of phospho-β1 integrin (pS785) levels. Pi3k, pAkt and Cdk1(pY15) levels are down-regulated by fibronectin and laminin I. Total Akt levels are not changed. ATDC5 cells are incubated in the presence of extracellular matrix molecules: fibronectin, laminin, and collagen, which serve as ligands for the β1 integrin receptor, and activation of the downstream pathways are assessed by Western blot analyses. Con = control, Col = collagen, Fib = fibronectin, and Lam = laminin. (G) Pretreatment of ATDC5 cells with Akt inhibitor VIII decreases Akt(pS473), Cdk1(pY15) and Sox9 levels, but increases protein levels of hypertrophic markers such as Runx2 and Col0a1. * = p<0.05, ** = p<0.01, *** = p<0.001; ^##^ = p<0.01, ^###^ = p<0.001. Star sign (*) represents comparison with control groups. (^#^) represents comparison between FlnA knockdown groups and FlnB knockdown groups. Scale bars = 200 µm, in A and B.

Recognizing the changes and the contribution of Cdk1 activity to the loss of FlnB phenotypes, we next asked whether there was a potential mechanistic link between FlnB and Cdk1. β1 integrin is a well known FlnA/B interactor, which also plays key roles in regulating bone development [27], [28], and our prior report had shown that phospho-β1 integrin (pS785) was downregulated in filamin B knockout chondrocytes [6]. We had also previously shown that FlnB could bind β1 integrin and loss of FlnB function led to downregulation of phospho-β1 integrin at pS785 [6]. In the current work, we found that phospho-β1 integrin at pS785 was dramatically decreased in the proliferating, prehypertrophic and hypertrophic zones by immunostaining *in vivo*. In stable null FlnB ATDC5 lines, we also found a reduction of protein expression for phospho-β1 integrin at pS785 by western blotting, consistent with our prior findings (Fig. 7A, B and C). Finally, phospho-Cdk1 levels were decreased in the FlnB knockdown cell lines suggesting that loss of FlnB could also indirectly effect Cdk1 activation. These observations suggested that FlnB could influence integrin and Cdk1 activity.

The extracellular signal related kinases (Erks) and phosphoinositide 3-kinase/protein kinase B (Pi3k/Akt) pathways play key roles in regulating bone development, function under β1 integrin signaling, and have been implicated in the regulation of Cdk1 [29], [30], [31], [32]. We therefore tested whether phosphorylated Erk levels (T202/Y204), p85 subunit of Pi3k, total and phosphorylated Akt (pS473) were changed in FlnB shRNA knockdown ATDC5 cells. Our results showed that phosphorylated Akt (pS473) but not total Akt levels were down-regulated with loss of FlnB (Fig. 7C, E). However, phosphorylated Erk levels did not change (Fig. 7C, E). Thus FlnB regulates Akt but not Erk activation (i.e. phosphorylation).

To further address whether Akt or Erk was involved under β1 integrin signaling, we seeded control ATDC5 cells onto pre-coated dishes with known β1 integrin activators such as collagen I, fibronectin and laminin type I and then examined both Akt/Erk changes as well as Cdk1 activation. Our results showed that fibronectin and laminin type I could induce down-regulation of Pi3k/Akt activity (no changes in total Akt), but did not induce significant changes in Erk activity (Fig. 7D, F). Cdk1 phosphorylation levels were also decreased following exposure to the extracellular matrix molecules, suggesting that integrin could mediate Cdk1 activity, possibly through a Pi3k/Akt pathway.

We next tested whether Erk or Pi3k/Akt signaling could induce Cdk1 activity changes in ATDC5 cells, as well as alter the differentiation state of the proliferating chondrocytes. The Erk activator TPA and Erk inhibitor U0126 were added into ATDC5 cell cultures but no significant Cdk1 activity changes were observed in either group (Supplementary Material, Fig. S6). However, the pAkt inhibitor VIII could not only induce downregulation of phospho-Cdk1(pY15), but also induced down-regulation of Sox9 levels and up-regulation of chondrocyte differentiation markers Runx2 and Col10a1 (Fig. 7G). Collectively, these studies suggested that Pi3k/Akt pathway mediated the β1 integrin signaling to the Cdk1 activity. Moreover, FlnB regulation of β1 integrin levels would suggest that FlnB could also indirectly influence Cdk1 activity through this pathway.

## Discussion

Our current work demonstrates that FlnB regulates Cdk1 phosphorylation levels, potentially through extracellular matrix mediated activation of β1 integrin and Pi3k/Akt. Cdk1 phosphorylation oversees proliferating chondrocyte progression through the G2/M phase of the cell cycle and differentiation. Thus, the thickened prehypertrophic zone, seen with loss of FlnB, reflects premature differentiation of proliferating chondrocytes. This premature differentiation also leads to a decrease in proliferation, a progressive decline in chondrocyte production over time, and ultimately a delay in bone growth. These findings provide a mechanistic explanation for the seemingly opposite phenotypes of early differentiation but delayed skeletal formation.

### Filamin B Regulates Chrondrocyte Proliferation and Long Bone Growth

FlnB loss of function in mice causes shortening of the skeletal appendicular bones and a delay in skeletal development over time [5], [6], [7], [8]. The current studies suggest that a progressive decline in the rate of proliferation may be responsible for limiting bone growth. We observed a decrease in various proliferation markers over time, including BrdU, Ki67 and PH3 [33], [34], [35]. The loss of proliferating cell numbers could extend from either prolongation of the cell cycle, early differentiation and/or increased cell death. TUNEL staining of null FlnB growth plates, however, did not suggest an increase in the number of apoptotic proliferating chondrocytes (data not shown). *FlnB*-knockdown ATDC5 proliferating chondrocytes did exhibit slower rates of proliferation with an apparent increase of G1/G0 phase subpopulations and a decrease of G2/M phase subpopulations, suggesting that more cells remained in G1/G0 phase and were undergoing differentiation. Accordingly, we observed early onset differentiation as evidenced by a decline in expression of earlier markers and increase in more mature chondrocyte markers. Moreover, chondrocytes with knockdown of FlnB showed downregulation of G2/M phase cell cycle markers, including Cyclin B1 and Cdc20 [36], [37], [38], [39]. These observations would indicate that increased maturation of the proliferating chondrocytes leads to slower proliferative rates and hence reduced chondrocyte production. In short, slower proliferation through increased chondrocyte maturation at least partially contributes to the observed skeletal phenotype.

### Loss of FlnB Promotes Chondrocyte Differentiation

FlnB loss of function can cause bone fusion accompanied by enhanced chondrocyte hypertrophy and premature differentiation [5], [6], [7], [8]. Loss of FlnB leads to premature differentiation with a widening of the prehypertrophic zone, as evidenced by an increase in immunostaining for the prehypertrophic (Pthr1 and Ihh) markers. We also observed an increase in overlap in the number of chondrocytes staining for both the proliferative (Col2a1) and hypertrophic (Col10a1) zone markers in FlnB^−/−^ mice, indicating some disruption in this intermediate stage of differentiation. The relative decrease in Col10a1 vs Col2a1 lengths with loss of FlnB would also argue that the greater defect lies in the transition from the proliferative to prehypertrophic zones, although these studies did not address whether there was any dysregulation in chondrocyte differentiation from the prehypertrophic to hypertrophic zones. That said, the FlnB knockdown ATDC5 cells showed characteristics of more terminal hypertrophic differentiation with increases in Col10a1, accompanied by decreases in the prehypertrophic markers Pthr1 and Ihh, when compared to the *in vivo* data. This observation indicates that chondrocytes ultimately undergo either normal or enhanced hypertrophic differentiation. Given that the ATDC5 cells do not undergo mineralization, the increase in hypertrophic phenotype could merely reflect this progressive differentiation. Somewhat surprisingly, our data show that the alkaline phosphatase activity was decreased rather than increased in the FlnB knockdown ATDC5 cells, in contrast to the upregulation of Col10a1 and Runx2. Given that alkaline phosphatase is a mineralization marker, the downregulation of alkaline phosphatase activity was actually consistent with our finding of a delay in bone formation. These results would suggest that FlnB promotes partial but not full chondrocyte hypertrophy. Overall, the accelerated premature differentiation seems to cause a slowing in the proliferative rate of the proliferating chondrocyte pool in the growth plate and ultimately delay bone growth.

### FlnB Regulates Chondrocyte Proliferation and Differentiation through Cell Surface Receptors

Two primary cell surface receptors have been implicated in FlnB-dependent regulation of chondrocyte differentiation. Recent work suggested that loss of FlnB leads to accumulation of phospho-Smad3 (through activation of the transforming growth factor beta receptor 1, Tgfbr1) [7]. Phospho-Smad3 promotes Runx2 function and early chondrocyte differentiation [7]. However, loss of TGF-β–Smad3 signaling also promotes chondrocyte hypertrophy, suggesting other regulators of this pathway. Prior studies from our laboratory and others have also shown that FlnB binds β1 integrin. Loss of β1 integrin function in chondrocytes leads to a progressive delay in chondrocyte differentiation within the hypertrophic zone and widening of prehypertrophic zone. Filamins play crucial roles in integrin regulation by either inhibiting integrin activation through C terminal binding or promoting integrin trafficking to plasma membrane for cell spreading [40]. These findings would indicate a more fundamental role for filamins near the cell surface in the regulation of various receptors and indirectly regulate the cell cycle and proliferation.

Prior reports have implicated FlnB-Smad3 interactions in inhibiting the downstream transcription factor Runx2 and promoting the null *FlnB* skeletal phenotype [7]. Runx2 promotes chondrocyte hypertrophy, and inhibition of Runx2 in the *FlnB^−/−^* mice appears to rescue some of the aberrant mineralization due to premature chondrocyte hypertrophy. However, inhibition of Runx2 still does not rescue the overall skeletal length, suggesting that Runx2 might play a partial causal role [7]. Smad 2,3 has previously been shown to regulate Cyclin D/E (regulators of G1 to S phase transition) whereas β-integrins mediate Cyclin B (regulators of G2/M) and D/E. Both Akt and Runx2 have been implicated in regulation of Cyclin B/D activity. The current studies indicate that FlnB-β1integrin interactions enhance activation of the Pi3k/Akt pathway, which promotes endochondral bone growth. Our studies would suggest that Akt directs CyclinB/Cdk1 dependent progression through G2/M phase. In this respect, FlnB likely binds and regulates activation of receptors (such as Smad and integrins) near the cell surface [6], [7], [32], [41]. These receptors mediate downstream mechanisms (Runx2 and/or Pi3k/Akt), which regulate chondrocyte proliferation and differentiation through cyclin-associated proteins. Phospho-Akt (pS473) changes were not observed in FlnB^−/−^ fibroblasts [8] suggesting that these changes might be cell specific or might reflect the observation that FlnB is preferentially expressed in bone (unpublished observations).

### Mechanisms Underlying FlnA/B in Regulation of Cell Cycle

The mechanisms by which filamins regulate CyclinB-Cdk1 activity in proliferating chondrocytes are likely complex. Our prior work has suggested that FlnA physically interacts with several Cyclin B associated proteins (such as Wee1) [12]. Moreover, FlnA impairs the degradation of these proteins that directly regulate Cdk1 phosphorylation. The current studies indicate that FlnB influences Cdk1 activity more indirectly than directly through its interactions with integrins at the cell surface. Integrin activation leads to downstream inactivation of various kinases (such as Akt) which direct Cdk1 function [29], [31], [32]. Finally, FlnA physically binds to FlnB to form heterodimers and this interaction likely influences both the dynamic activation of receptors at the cell surface and clearance of cyclin B associated proteins.

How FlnA and FlnB differentially mediate proliferation and development may be of some importance. We have recently shown that loss of FlnA results in an increased number of cells residing in G2/M phase, and to a lesser degree, in G1/S phase- thereby leading to a prolongation of the cell cycle and delayed differentiation [12]. The delay in progression through G2/M phase was due to impaired degradation of Wee1 (a Cyclin B-associated protein) and consequent increase in phospho-Cdk1. The current studies demonstrate that in some fashion, loss of FlnB leads to a directly opposite phenotype with fewer proliferating chondrocytes residing in G2/M phase (promoting cell cycle progression), and premature differentiation.

The current observations raise the question as to how the balance between FlnA and FlnB is tightly controlled and how their differential roles are integrated coordinately during development. Several studies have implicated FlnA in regulation of endosomal vesicle trafficking through the caveolin pathways, suggesting that stabilization or activation of cell surface receptors such as β1 integrin [32] or TGFβ1 (Smad) [41] may be dependent on the filamins. Alternatively, we have observed a role for FlnA in ubiquitination (data not shown) and filamins have been linked to E3 ligases in the ubiquitin pathway [42], raising the possibility that these proteins could mediate the clearance of the cyclin associated proteins. In this respect, FlnA may enhance degradation of specific cell cycle proteins whereas FlnB may antagonize this role. Future studies will be required to evaluate the relationship between FlnA and FlnB in regulating these pathways.

## Materials and Methods

### Ethics Statement

All mouse studies were performed under approval from the Institutional Animal Care and Use Committees of Harvard Medical School and Beth Israel Deaconess Medical Center in accordance with *The National Institutes of Health Guide for the Care and Use of Laboratory Animals*.

### FlnB^−/−^ Mice Breeding, BrdU Injections, Tissue Isolation and Bone Decalcification

The FlnB^−/−^ mice were generated and bred as previously reported [6]. The wild-type allele was detected by PCR amplification using the primer pair 5′-agattattcacccggacgtg-3′ and 5′-cctgggctaataatggcaga-3′, and the mutated allele by using 5′-ctgtgctcgacgttgtcactg-3′ and 5′- gatcccctcagaagaactcgt-3′. For the *in vivo* chondrocyte proliferation assays, E14.5, E16.5 pregnant mice or P7 newborn mice were injected with BrdU at 50 mg/kg ip. after plugging date, and the mice were sacrificed after 3 or 48 hours. Ketamine/Xylazine combination was used for anesthesia and euthanization (100 mg/kg and 10 mg/kg, 400 mg/kg and 40 mg/kg, respectively, injected intraperitoneally). Tissues were isolated after euthanization and fixed with 4% paraformaldehyde or 10% trichloroacetic acid. Additional samples were frozen, sectioned, and fixed prior to staining. Young and adult tissues were further decalcified by 5% trichloroacetic acid solution containing 1% HCl and 1% acetic acid for 7 days.

### Skeletal Staining, Routine Histology, Immunostaining and Imaging

Skeletal staining and routine histology, immunostaining of the radial bones was previously performed in this laboratory [6]. In brief, different age wild-type and mutant mice were euthanized as above, forearms dissected, skinned and eviscerated, and the skeleton was dehydrated in 95% ethanol overnight and acetone overnight. The radial bones were then stained with Alizarin red (0.005%) and Alcian blue (0.015%) in a solution containing ethanol, glacial acetic acid and water (60∶5∶35) at 37°C overnight. The stained embryos were then transferred to 1% potassium hydroxide solution for 7 days to dissolve the soft tissue. The skeletal bones were preserved in glycerol.

Conventional HE staining methods with mild modifications was used. In brief, bone tissues were fixed with 10% (w/V) trichloroacetic acid (TCA) for more than 24 hours and then decalcified with 5% TCA solution containing 1% HCl and 1% acetic acid for 7 days. 18 micron frozen sections were placed into water for 5 minutes, then stained with 1× Hematoxylin solution (Cat.# HHS32, Sigma, St. Louis, MO, USA) for 3 minutes. After water washing, sections were place in 3% acetic acid/70% ethanol v/v solution. After washing in water, samples were incubated with Scott’s solution for 30 seconds. After water washing and 95% ethanol incubation, sections were stained with alcoholic Eosin Y for 3 minutes. Finally, after serial dehydration through graded ethanol solutions, sections were rinsed in xylene and mounted with cytoseal 60 (Richard-Allan). Images were obtained with an Axioskop microscope (Zeiss, Germany).

For immunostaining of FlnB, Sox9, Pthr1, Col2a1, Col10a1, Runx2, Cdk1(pY15), Cyclin B1, Cdc20, Cdc25c, Wee1 and Pkmyt1, tissues and cells were fixed using 10% (w/V) ice-cold TCA for 20 minutes. For staining of other antibodies, the samples were fixed with 4% paraformaldehyde for 10 minutes. After washing in PBS, fixed samples were permeabilized with 0.5% Triton X-100 and blocked with 5% normal horse serum for 2 hours. Tissue was incubated with the primary antibodies for 1 hour at room temperature or overnight at 4°C. The Dylight488- and Dylight594-conjugated secondary antibodies (Jackson Immunoresearch, West Grove PA, USA) were incubated for 1 hour at room temperature. Samples were further counterstained with 100 ng/ml Hoechst33342 (Life Technologies, Grand Island, NY, USA). Images were obtained with an LSM5 Pascal confocal microscope (Zeiss, Germany). The staining intensity was analyzed by histogram signal intensity using Adobe Photoshop(each growth plate is divided into 30 fractions from the secondary germinal center to the border of the hypertrophic zone as labeled in each panel, and the signal intensity(luminosity) is determined with Adobe Photoshop Histogram Tool. The primary antibodies (for immunostaining and some also for western blotting) were: rabbit anti-FlnA monoclonal antibody (1∶300, Cat.# 2242, Epitomics, Burlingame, CA, USA); rabbit anti-FlnB polyclonal antibody (Gifted by Dr. Kao, CWRU); mouse anti-Col2a1 (Cat.# Ab3092, ABCAM, USA); rabbit anti-Col10a1 (kindly gifted by Dr. Horton and Dr. Lunstrum, Shriners Hospital for Children, Portland, OR, USA; [21]); rabbit anti-Pthr1 (Cat.# Ab75150, ABCAM, USA);rabbit anti-Ihh (Cat.# sc-13088, Santa Cruz); rabbit anti-Runx2 (Cat.# sc-10758, Santa Cruz); rabbit anti-Sox9 pab (1∶300, AB5535, Millipore); rabbit anti-Sox9 pab (O9-1, gift of Professor Dr. Michael Wegner, Institute of Biochemistry, Friedrich-Alexander-University, Erlangen-Nurnberg, Germany); rat anti-BrdU (1∶150, Cat.# MCA2060, AbD Serotec, Raleigh, NC, USA); rabbit anti-Ki-67 mab (1∶200, Cat.# 4203, Epitomics); rabbit anti-PH3 pab (1∶250, Cat:# 06-570, Millipore, Billerica, MA, USA); mouse and rabbit anti-Wee1 (1∶50, Cat.# sc-5285 and sc-325, Santa Cruz); rabbit anti-Pkmyt1 (1∶100, Cat.# 3303, Epitomics); anti-pan 14-3-3 (Santa Cruz, sc-629); mouse and rabbit anti-cyclin B1 (1∶100, Cat.# sc-245 and sc-752, Santa Cruz); mouse anti-Cdc20 (1∶100, Cat.# sc-13162, Santa Cruz); mouse and rabbit anti-cdc25c (1∶100, Cat.# sc-55513 and sc-327, Santa Cruz); rabbit-anti-Cdk1 (Cat.# PC25,Calbiochem, San Diego, CA, USA); mouse anti-Cdk1(pY15) (Cat.# BD612306, BD, Franklin Lakes, NJ USA); rabbit anti-Pi3k (p85 subunit alpha, Cat#: 1675, Epitomics); rabbit anti-Akt (phospho-S473, Cat# 4060, Cell Signaling); rabbit anti-Erk1/2 (phospho-T202/Y204, Cat.# 4370, Cell Signaling); rat anti-β1 integrin (Cat.# mab1997, Millipore); rabbit anti- β1 integrin(pS785) (Cat.# OPA1-03177, Affinity BioReagents). In situ hybridization studies for Ihh, Col2a1 and Col10a1 have been previously described [5], [6].

### Mouse Primary Chondrocyte Isolation and Cell Culture

Primary chondrocytes from growth plates of newborn mice were prepared according to the Lefebvre’s protocol [43] with mild modifications [6]. In brief, the growth plates of radius and ulna were dissected from P7 newborns, minced and washed in cold Hanks buffered saline solution, and placed in 0.2% collagenase type I (Cat.# 4196, Worthington, Lakewood, NJ, USA) solution in DMEM at 37°C for 3 hours. Soft tissues were detached from cartilage by repeated pipetting; the sediment cartilage was further digested with 0.2% collagenase I for 6 hours. The dissociated cells were then passed through a cell strainer to isolate single cells and were plated at feasible density in DMEM with 10% FBS.

### ATDC5 Cell Culture and Proliferation Analysis

Mouse ATDC5 chondrocyte cells (Cat# 99072806, Sigma) and shRNA expressing ATDC5 cells were cultured in DMEM medium containing 10% fetal bovine serum, 100 IU/ml penicillin, and 100 µg/ml streptomycin (Cat.# 15140, Invitrogen). Unless otherwise specified, ATDC5 cells were passaged every two days. For cell growth curve test, 1×10^5^ ATDC5 cells/well were seeded into 12-well plates and incubated for 6 days. Triplicate wells for each time points were used. Cell numbers were counted everyday. For BrdU incorporation test, 10 µM BrdU was added to the ATDC5 cells growing on cover slips for 1 hour and labeled with rat-anti-BrdU antibody.

### shRNA Vector Construction, Lentivirus Preparation and Infection

Two sets of shRNA target sequences were designed and corresponding oligomers were synthesized. The shRNA sequences were: shRNA1, 5′-gccgacattgaaatgccgttt-3′; shRNA2, 5′-gcccaaatcaagactcttaat-3′. After heating to 98°C for 10 minutes, the oligomers were reannealed in boiling water that gradually cooled back to room temperature, followed by gradual cooling back to room temperature. Then the annealed products were ligated into the HpaI/XhoI sites of the EGFP tagged pSicoR lentivirus vector. The helper vectors, pD8.9 and pVSVG were transfected together with the lentivirus vector into 293T cells. For the control group, empty pSicoR vector was used. The TransFectin Lipid reagent was used according to manufacturer’s instructions (Cat.# 170-3352, BIO-RAD). Lentivirus containing medium was collected by centrifugation at 2000×g for 10 minutes. Then the supernatant was passed through a 0.45 um filter. The filtered medium was directly added to the pre-seeded ATDC5 cells. The infected ATDC5 cells were visualized by microscope and were verified by confocal scanning. After two rounds of infection, all the ATDC5 cells were EGFP positive. The EGFP positive ATDC5 cells were used for further *in vitro* experiments. Several additional lines were generated in parallel fashion, including null FlnA lines which served as comparative controls.

### Cdk1 Inhibition, Akt Inhibition, β1 Integrin Activation, Erk Activation and Inhibition

A Cdk1 phosphorylation inhibitor (3-(2-Chloro-3-indolylmethylene)-1,3-dihydroindol-2-one, 50 mM) was dissolved in DMSO and added into cultured ATDC5 cells at final concentrations of 5 µM, 3 µM and 1 µM for titration. The Cdk1 inhibitor at 1 µM did not induce cell death in ATDC5 cells and this concentration was used for cell growth curve analysis and differentiation induction analysis. The Cdk1 inhibitor was added to cells and incubated for 6 days for cell growth curve analysis with daily quantification of cell numbers. For differentiation induction analyses, the Cdk1 inhibitor was added and cells were incubated for 6 days, with the cells being passaged every two days. For β1 integrin activation, culture dishes were pre-coated with Collagen I (cat#: 354236, BD Biosciences), fibronectin (F0895, Sigma) and laminin type I (Cat#: 354232, BD Biosciences) following manufacture’s instructions. ATDC5 cells were incubated for 4 hours and 48 hours respectively and were collected for western blotting analysis. Erk activator TPA (12-O-Tetradecanoylphorbol-13-Acetate, 200 nM, Cell Signaling) and Erk inhibitor U0126 (10 µM, Cell Signaling) were added into cultured ATDC5 cells for 30 minutes and 24 hours respectively for Erk and Cdk1 activity analysis. Akt inhibitor VIII (Isozyme-Selective, Akti-1/2, Cat# 124018, Millipore) was dissolved in DMSO and added into cultured ATDC5 cells at final concentration of 10 µM for 48 hours for Akt(pS473), Cdk1(pY15) and differentiation analysis. The same volumes of DMSO were added as controls for signaling activation and inhibition tests.

### RT-PCR

Total RNA samples were prepared from ATDC5 cells and shRNA expressing ATDC5 cells. The RNA was extracted from adult tissues using TRIzol (Invitrogen). Reverse transcription and PCR were performed as conventional methods by using SuperScript III First Strand Kits (Cat# 18080-400, Invitrogen). The cDNA was amplified by PCR for 30 cycles. Primers were selected encompassing the intron sequences. The primer sequences used for *FlnB* RT-PCR analysis were: forward, 5′-tcttcccacatacgatgcaa-3′; reverse, 5′-tccactacaaagcccacctc-3′. The *Gapdh* was used as internal control and the primers were: forward, 5′-ggcaaagtggagattgttgcc-3′; reverse, 5′-aagatggtgatgggcttcccg-3′. PCR cycling was performed at 95°C for 2 minutes followed by 95°C for 30 seconds, 55°C for 30 seconds, 72°C for 1 minutes, and finally 72°C for 6 minutes, by using the EmeraldAmp MAXPCR master mix (Takara, Cat.# RR320A, Japan). PCR products were analyzed by 1.5% agarose gel.

### Immunoprecipitation and Western Blotting

Samples were collected either immediately or 36 hours after transfection. Modified RIPA buffer (50 mM Tris-HCl, pH7.5; 150 mM NaCl, 1.0% Triton x-100; 0.5% sodium deoxycholate and 0.1% SDS), with proteinase inhibitor cocktail and protein phosphotase inhibitor cocktail, as well as additional NaF (10 mM) and Na3VO4 (1 mM), was used for cell lysis. Conventional immunoprecipitations were performed by using Protein-A/G ultralink resin beads according to the protocol provided by the manufacturer (Cat.# 53132, Thermo Scientific, Rockford, IL, USA Proteins were separated in 8% or 10% SDS-polyacrylamide gels and transblotted to PVDF membrane. Immunoblot analysis was performed with primary antibodies based on manufactures’ guides or suggested dilutions. Blots were detected by using LumiGOLD ECL western blotting detection kit (Cat.# SL100309, SignaGen, Rockville, MD, USA).

### Alkaline Phosphatase Assay

Alkaline phosphatase activity was determined by using alkaline phosphatase assay kit (Cat# ab83369, ABCAM) following manufacture’s instructions. In brief, cultured control and FlnB knockdown ATDC5 cells (1×10^6^) were homogenized with assay buffer after cold PBS washing. For each sample, 10, 20, 30, 40 and 50 µl of supernatant were added into triplet 96-wells and each well was brought to a total volume of 80 µl with assay buffer. Triplets of 30 µl of each sample were added into 96-wells and 50 µl of assay buffer was added, followed by adding 20 µl of stop-solution for background control. Standard curves were generated as manufacture’s guide. The alkaline phosphatase enzyme activity of each sample was calculated based on the comparison between standard curve and sample curve, and was shown as Unit/ml (U/ml).

### Statistical Analyses

Results were expressed as the mean±s.d. of n experiments (n≥3). For section staining, unless otherwise specified, 3 animals were used for each experiment and 3–4 sections were stained per animal. For cultured cell staining, cells were plated and growing on 3 coverslips per sample. For cell growth curve analysis, 3 independent experiments were performed, with 3–4 wells were collected from each sample at each time point. Statistical analysis was performed with Student’s t-test, with P<0.05 considered significant. For quantitative analyses of immunostaining results, luminosity (histogram value, Adobe photoshop) values were obtained and based on the threshold (luminosity value 20) cells were grouped into highly stained and weakly/negatively stained subpopulations. For quantitative analyses of western blotting results, band intensity was obtained by subtracting background luminosity from the total luminosity of each band (histogram value, Adobe photoshop).

## Supporting Information

Figure S1 **FlnB knockdown efficiency in ATDC5 chondrocyte progenitors.**
(DOC)

Figure S2 **FlnB^−/−^ mice display skeletal shortening and abnormalities in the growth plates of the appendicular bones.**
(DOC)

Figure S3 **FlnB^−/−^ mice display reduced Col10a1^+^/Col2a1^+^ ratio in the growth plates of the appendicular bones.**
(DOC)

Figure S4 **Increased differentiation with loss of FlnB function in the long bone.**
(DOC)

Figure S5 **Diminished expression of Cyclin B-associated proteins in FlnB knockdown chondrocyte progenitors.**
(DOC)

Figure S6 **Erk activation and inhibition assay.**
(DOC)
