# Supplementary material for: Filamin B Regulates Chondrocyte Proliferation and Differentiation through Cdk1 Signaling
Source: PLoS One. 2014 Feb 14;9(2):e89352. doi: 10.1371/journal.pone.0089352 (PMC3925234; doi:10.1371/journal.pone.0089352)
Supplement: Figure S1 — FlnB knockdown efficiency in ATDC5 chondrocyte progenitors. (DOC) [file pone.0089352.s001.doc]

**
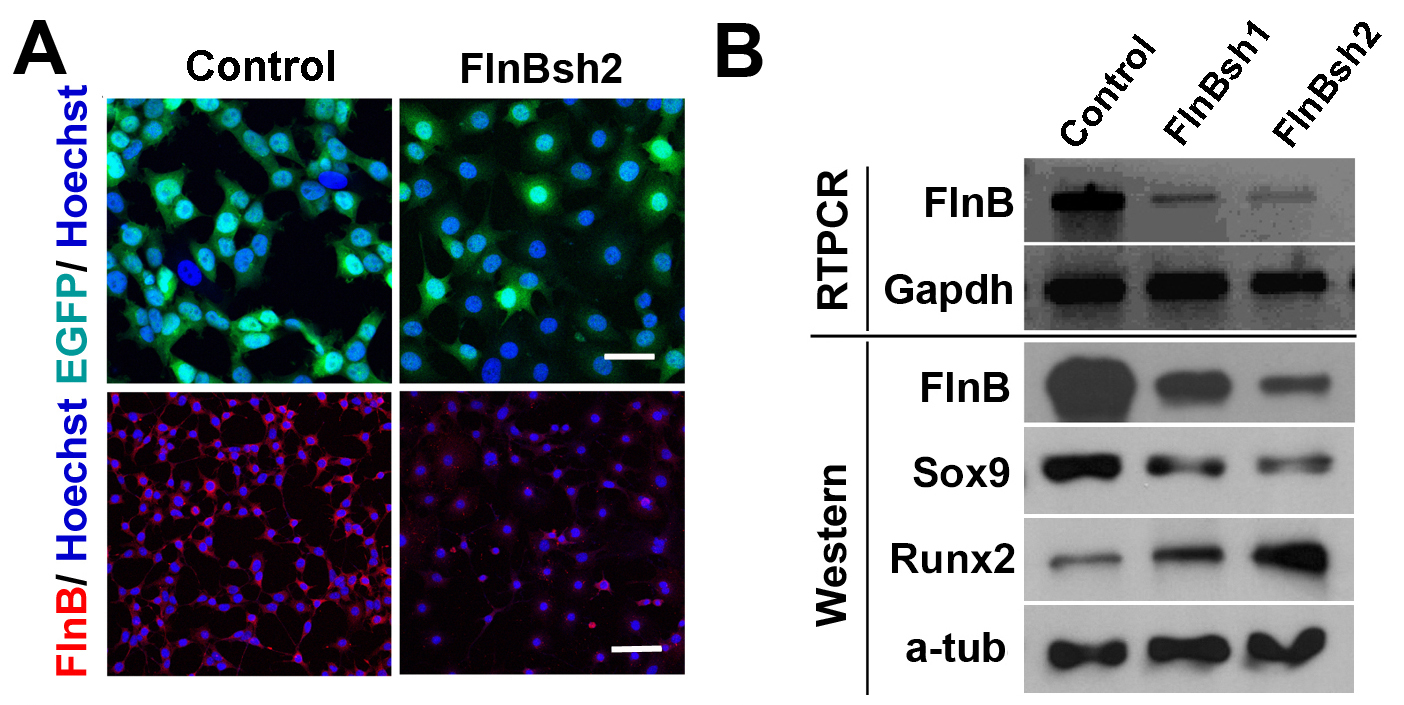
**

**Figure S1. FlnB knockdown efficiency in ATDC5 chondrocyte progenitors.** (**A**) Fluorescent confocal photomicrographs (fluoroscein) show stable transfection of either EGFP alone (control) or EGFP-FlnB shRNA (FlnBsh2) cells grown in culture after prolonged culturing. Rhodamine fluorescence demonstrates down regulation of FlnB expression in the FlnBsh2 cell line. (**B**) FlnB knockdown leads to decreased mRNA and protein expression for FlnB as evidenced by RT-PCR and western blot analyses. Sox9 a marker of premature proliferating chondrocytes is down-regulated by knocking down FlnB whereas Runx2, a marker for chondrocyte differentiation is up-regulated. Scale bar=50 μm in A upper panel and 100 μm in A lower panel.
