# Supplementary material for: Filamin B Regulates Chondrocyte Proliferation and Differentiation through Cdk1 Signaling
Source: PLoS One. 2014 Feb 14;9(2):e89352. doi: 10.1371/journal.pone.0089352 (PMC3925234; doi:10.1371/journal.pone.0089352)
Supplement: Figure S2 — FlnB−/− mice display skeletal shortening and abnormalities in the growth plates of the appendicular bones. (DOC) [file pone.0089352.s002.doc]

**
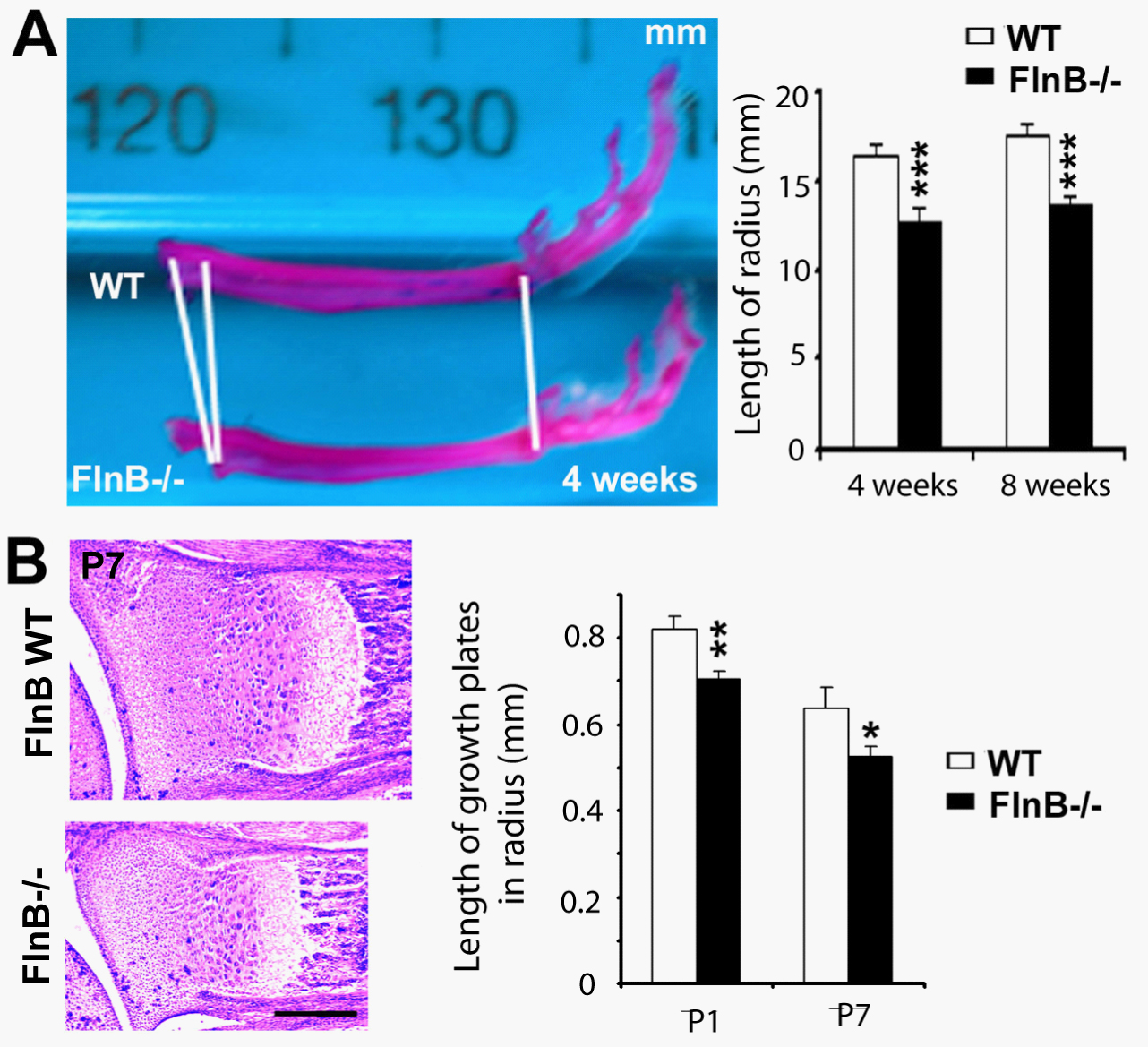
**

**Figure S2. FlnB^-/-^ mice display skeletal shortening and abnormalities in the growth plates of the appendicular bones.** (**A**) Gross specimen of 4 week old radius from *FlnB^-/-^*and control mice following Alizarin red (calcified bone) and Alcian blue (cartilage) staining. The forearm length is reduced with FlnB knockout and quantified to the right. (**B**) Hematoxylin and eosin (H&E) staining reveals that the reduction in bone length in the radius of P1 and P7 FlnB^-/-^ mice is due to thinning of the growth plates compared to wild type, consistent with our prior published observations of shortening at embryonic ages. Quantification at these postnatal ages is graphically shown to the right. For all experiments, n samples >=3 per variable, *= p< 0.05, **=p<0.01, ***= p <0.001 by t-test. Scale bar= 200 μm for B.
