# Supplementary material for: Filamin B Regulates Chondrocyte Proliferation and Differentiation through Cdk1 Signaling
Source: PLoS One. 2014 Feb 14;9(2):e89352. doi: 10.1371/journal.pone.0089352 (PMC3925234; doi:10.1371/journal.pone.0089352)
Supplement: Figure S3 — FlnB−/− mice display reduced Col10a1+/Col2a1+ ratio in the growth plates of the appendicular bones. (DOC) [file pone.0089352.s003.doc]

**
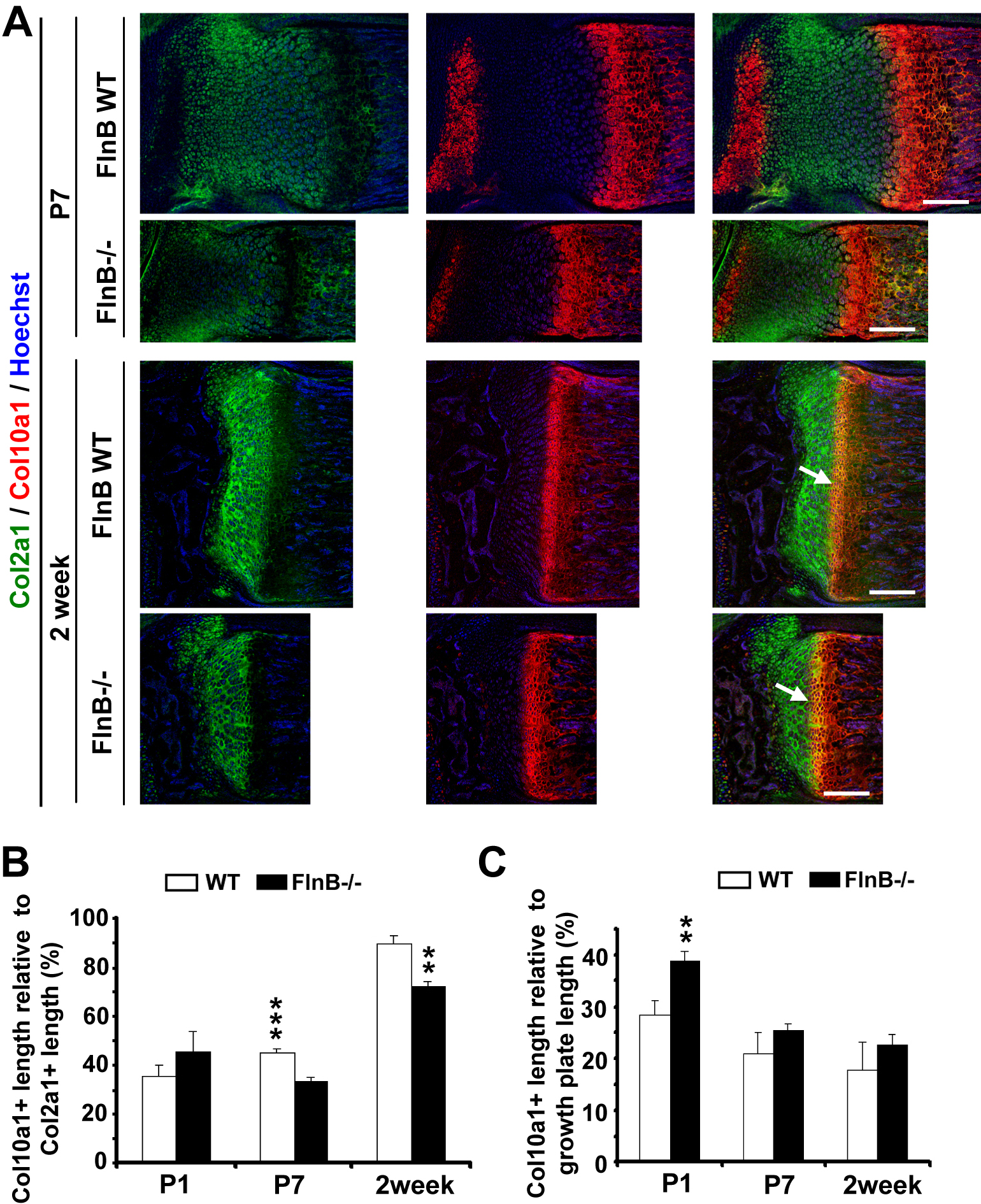
**

**Figure S3. FlnB^-/-^ mice display reduced Col10a1^+^/Col2a1^+^ ratio in the growth plates of the appendicular bones.** (**A**) Double immunostaining for Collagen2 (Col2a1) and Collagen10 (Col10a1) on p7 and 2 week old radius of null FlnB mice (Please see Figure 1A for P1 age). The arrows indicate the overlapping region of expression between Col2a1 and Col10a1 is increased in the null FlnB mice. (**B**) Quantitative analysis of the Col10a1^+^ length relative to Col2a1^+^ length. The ratio of Col10a1^+^ length relative to Col2a1^+^ length (hypertrophic to proliferative zone ratio), is decreased in FlnB^-/-^ mice from P7 to 2 weeks age (33.8% vs 45.3% and 71.9% vs 89.6% in *FlnB^-/^*^-^ and wild type, respectively). The ratio at P1 age does not show significant change. (**C**) Quantitative analysis the relative thickness of the Col10a1^+^ hypertrophic zone (Col10a1^+^/whole growth plate) is increased at this age. **=p<0.01, ***= p <0.001 by Student’s t-test. Scale bars= 200 μm.
