# Supplementary material for: Filamin B Regulates Chondrocyte Proliferation and Differentiation through Cdk1 Signaling
Source: PLoS One. 2014 Feb 14;9(2):e89352. doi: 10.1371/journal.pone.0089352 (PMC3925234; doi:10.1371/journal.pone.0089352)
Supplement: Figure S4 — Increased differentiation with loss of FlnB function in the long bone. (DOC) [file pone.0089352.s004.doc]

**
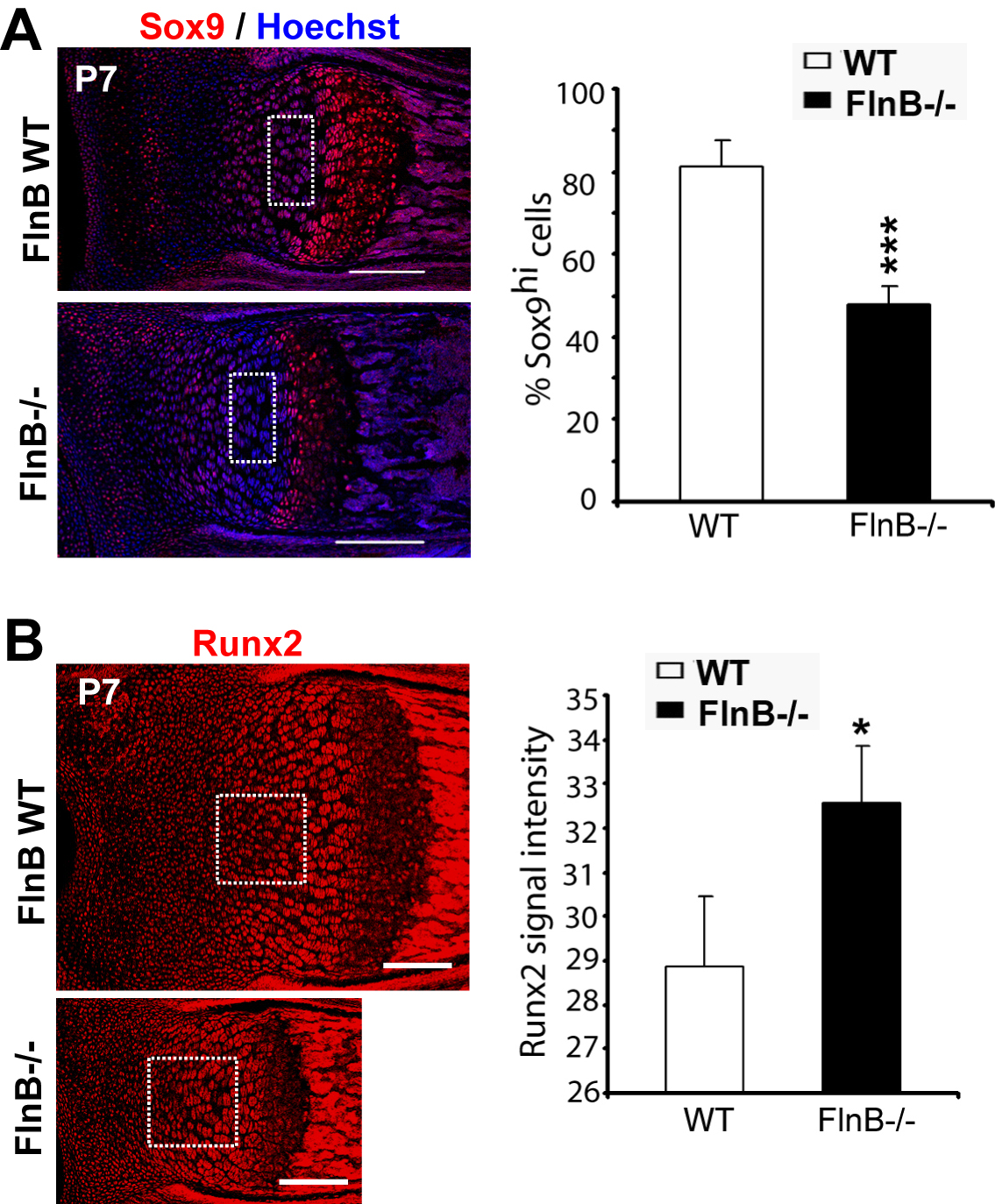
**

**Figure S4. Increased differentiation with loss of FlnB function in the long bone.** (**A**) Immunostaining of the long bones at P7 shows that the intensity of Sox9 is dramatically decreased in *FlnB^-/-^* mice, especially in the rapid proliferative zone and prehypertrophic zone. (**B**) Immunostaining for Runx2, a chondrocyte marker that is up-regulated during both endochondral and intramembranous ossification, is increased within chondrocyte progenitors within the proliferative zone, following FlnB knockout. For all experiments, n samples >=3 per variable, *= p< 0.05, **=p<0.01, ***= p <0.001 by t-test. Scale bars= 200 μm.
