# Supplementary material for: Filamin B Regulates Chondrocyte Proliferation and Differentiation through Cdk1 Signaling
Source: PLoS One. 2014 Feb 14;9(2):e89352. doi: 10.1371/journal.pone.0089352 (PMC3925234; doi:10.1371/journal.pone.0089352)
Supplement: Figure S5 — Diminished expression of Cyclin B-associated proteins in FlnB knockdown chondrocyte progenitors. (DOC) [file pone.0089352.s005.doc]

**
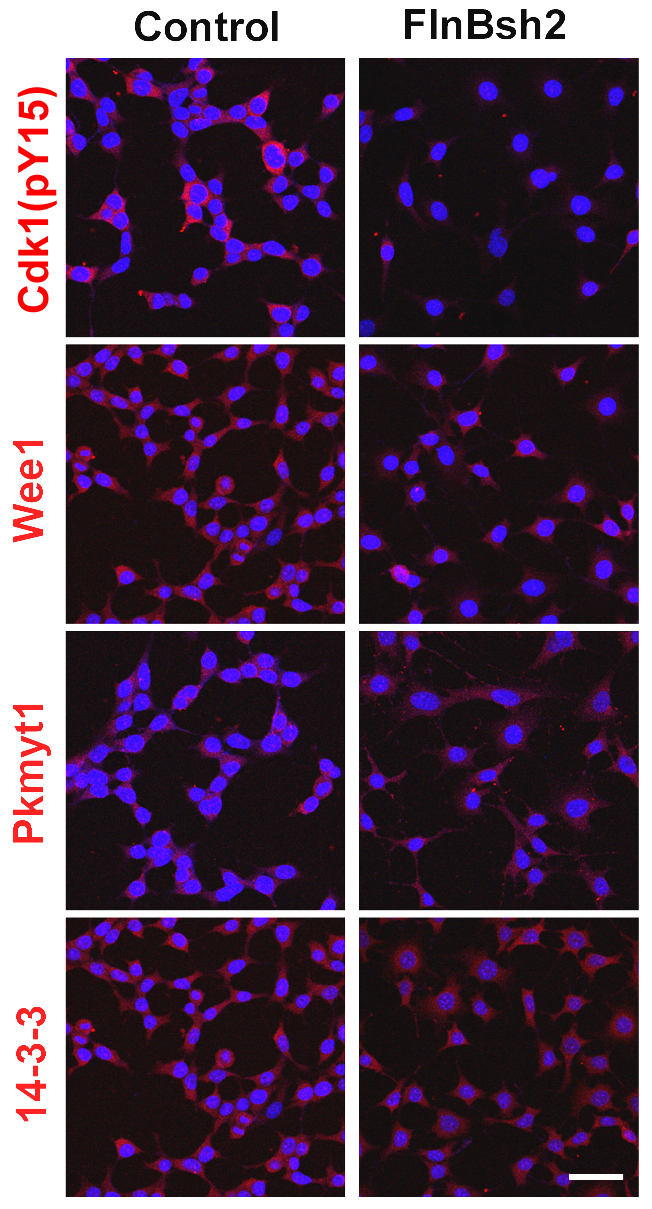
**

**Figure S5. Diminished expression of Cyclin B-associated proteins in FlnB knockdown chondrocyte progenitors.** Fluorescent confocal photomicrographs shows downregulation of various Cyclin B1-associated proteins including phosphorylated Cdk1(pY15) and Wee1 (red), following loss of FlnB function in ATDC5 stable cell lines. Pkmyt1 and 14-3-3 do not show dramatic changes with loss of FlnB on immunostaining, but their expression is diminished by western blot analyses. Diminished expression of these proteins following loss of FlnB expression are largely consistent with the G2/M phase changes seen in the FlnBsh2 cell line and the observation of decreased proliferation and increased differentiation within the chondroprogenitor pool. Scale bar=50 μm.
