# Supplementary material for: Filamin B Regulates Chondrocyte Proliferation and Differentiation through Cdk1 Signaling
Source: PLoS One. 2014 Feb 14;9(2):e89352. doi: 10.1371/journal.pone.0089352 (PMC3925234; doi:10.1371/journal.pone.0089352)
Supplement: Figure S6 — Erk activation and inhibition assay. (DOC) [file pone.0089352.s006.doc]

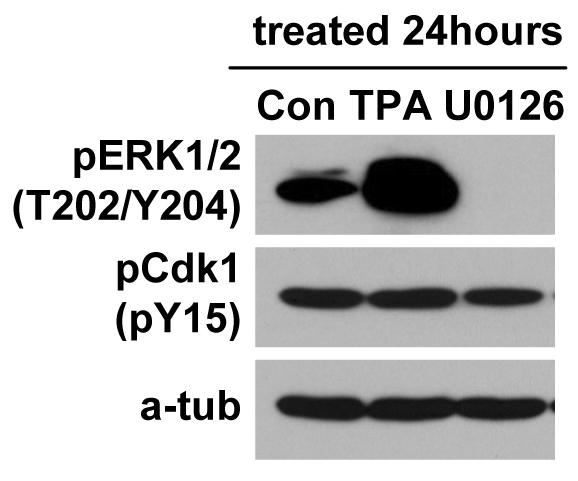


**Figure S6. Erk activation and inhibition assay.** Erk activator TPA (12-O-Tetradecanoylphorbol-13-Acetate, 200 nM, Cell Signaling) and Erk inhibitor U0126 (10 μM, Cell Signaling) were added into cultured ATDC5 cells for 30 minutes (data not shown) and 24 hours for Erk and Cdk1 activity analysis, respectively. Erk Activation by TPA or inhibition by U0126 do not induce significant Cdk1(pY15) changes in ATDC5 cells.
